# Plasmolipin regulates basolateral-to-apical transcytosis of ICAM-1 and leukocyte adhesion in polarized hepatic epithelial cells

**DOI:** 10.1007/s00018-021-04095-z

**Published:** 2022-01-09

**Authors:** Cristina Cacho-Navas, Natalia Reglero-Real, Natalia Colás-Algora, Susana Barroso, Gema de Rivas, Kostantinos Stamatakis, Jorge Feito, Germán Andrés, Manuel Fresno, Leonor Kremer, Isabel Correas, Miguel A. Alonso, Jaime Millán

**Affiliations:** 1 Centro de Biología Molecular Severo Ochoa, Universidad Autónoma de Madrid, CSIC-UAM, Cantoblanco, 28049 Madrid, Spain; 2 Servicio de Anatomía Patológica, Hospital Universitario de Salamanca, Salamanca, Spain; 3 William Harvey Research Institute, Barts and the London School of Medicine and Dentistry, Queen Mary University of London, London, UK; 4 Department of Immunology and Oncology, Centro Nacional de Biotecnología, CNB-CSIC, Madrid, Spain; 5 Protein Tools Unit, Centro Nacional de Biotecnología, CNB-CSIC, Madrid, Spain

**Keywords:** ICAM-1, PLLP, Hepatocyte, Apicobasal polarity, Lymphocyte adhesion, Transcytosis, Subapical compartment, bile canaliculus, BioID

## Abstract

Apical localization of Intercellular Adhesion Receptor (ICAM)-1 regulates the adhesion and guidance of leukocytes across polarized epithelial barriers. Here, we investigate the molecular mechanisms that determine ICAM-1 localization into apical membrane domains of polarized hepatic epithelial cells, and their effect on lymphocyte-hepatic epithelial cell interaction. We had previously shown that segregation of ICAM-1 into apical membrane domains, which form bile canaliculi and bile ducts in hepatic epithelial cells, requires basolateral-to-apical transcytosis. Searching for protein machinery potentially involved in ICAM-1 polarization we found that the SNARE-associated protein plasmolipin (PLLP) is expressed in the subapical compartment of hepatic epithelial cells in vitro and in vivo. BioID analysis of ICAM-1 revealed proximal interaction between this adhesion receptor and PLLP. ICAM-1 colocalized and interacted with PLLP during the transcytosis of the receptor. *PLLP* gene editing and silencing increased the basolateral localization and reduced the apical confinement of ICAM-1 without affecting apicobasal polarity of hepatic epithelial cells, indicating that ICAM-1 transcytosis is specifically impaired in the absence of PLLP. Importantly, PLLP depletion was sufficient to increase T-cell adhesion to hepatic epithelial cells. Such an increase depended on the epithelial cell polarity and ICAM-1 expression, showing that the epithelial transcytotic machinery regulates the adhesion of lymphocytes to polarized epithelial cells. Our findings strongly suggest that the polarized intracellular transport of adhesion receptors constitutes a new regulatory layer of the epithelial inflammatory response.

**Supplementary Information:**

The online version contains supplementary material available at 10.1007/s00018-021-04095-z.

## Introduction

The type I transmembrane protein intercellular adhesion molecule (ICAM)-1 is the counterreceptor of leukocyte β2-integrins and mediates the firm adhesion of leukocytes to epithelial and endothelial cells [1–3]. ICAM-1 is apically confined in polarized intestinal and hepatic epithelia [2, 4, 5]. Such polarization confers on these cellular barriers the capacity to establish a haptotactic gradient between apical and basolateral membrane domains, which guides infiltrated immune cells [2, 6]. In hepatocytes and cholangiocytes, apical membrane domains form tubular networks, respectively, known as bile canaliculi and bile ducts, which drain bile acids and other hepatic molecules into the gastrointestinal tract. ICAM-1 is mostly confined in these apical structures and is therefore not accessible to circulating immune cells, which preferentially adhere to hepatic epithelial cells when they lose their polarity and expose the receptor [5]. This ability of epithelial apicobasal polarity to regulate leukocyte adhesion potentially allows the immune system to discriminate between polarized, operative cells and depolarized, dysfunctional hepatic epithelial cells.

The molecular bases determining the apical localization of ICAM-1 in epithelial cells are currently unknown, but involve removing ICAM-1 from the basolateral membrane domains and translocating the receptor to apical bile canalicular-like structures (BCs) [5]. This basolateral detour to BCs has been observed for other type I transmembrane proteins [7] suggesting that it is a general mechanism of apical sorting of proteins with this topology. The MAL family of integral proteins is involved in polarized vesicular trafficking [8, 9] and in the organization of liquid-ordered membrane domains [10, 11]. MAL proteins contain at least one MARVEL domain and are differentially expressed, playing specialized functions in the intracellular transport of proteins in various cell types [8, 9, 12]. The MAL protein plasmolipin (PLLP) is a proteolipid that is strongly expressed in oligodendrocytes and localized in myelin [13] and also sorts endosomal SNAREs to the subapical compartment (SAC) of polarized renal and intestinal epithelial cells, where it determines the localization of the type I transmembrane protein Crumbs, which is involved in epithelial morphogenesis and apicobasal polarity [14].

Given the importance of ICAM-1 polarity in different epithelial beds, we have investigated the mechanisms mediating basolateral-to-apical trafficking of ICAM-1 and identified PLLP as a part of the intracellular machinery that mediates basolateral-to-apical transport of ICAM-1 from the SAC in hepatic epithelial cells. Importantly, epithelial PLLP expression controls ICAM-1-mediated adhesion of leukocytes to polarized hepatic cells, revealing a new role for the transcytotic machinery in regulating the epithelial inflammatory response.

## Results

### PLLP is expressed in the SAC of polarized hepatic epithelial cells and interacts with ICAM-1 during the basolateral-to-apical transcytotic route of the receptor

**Fig. 1:**
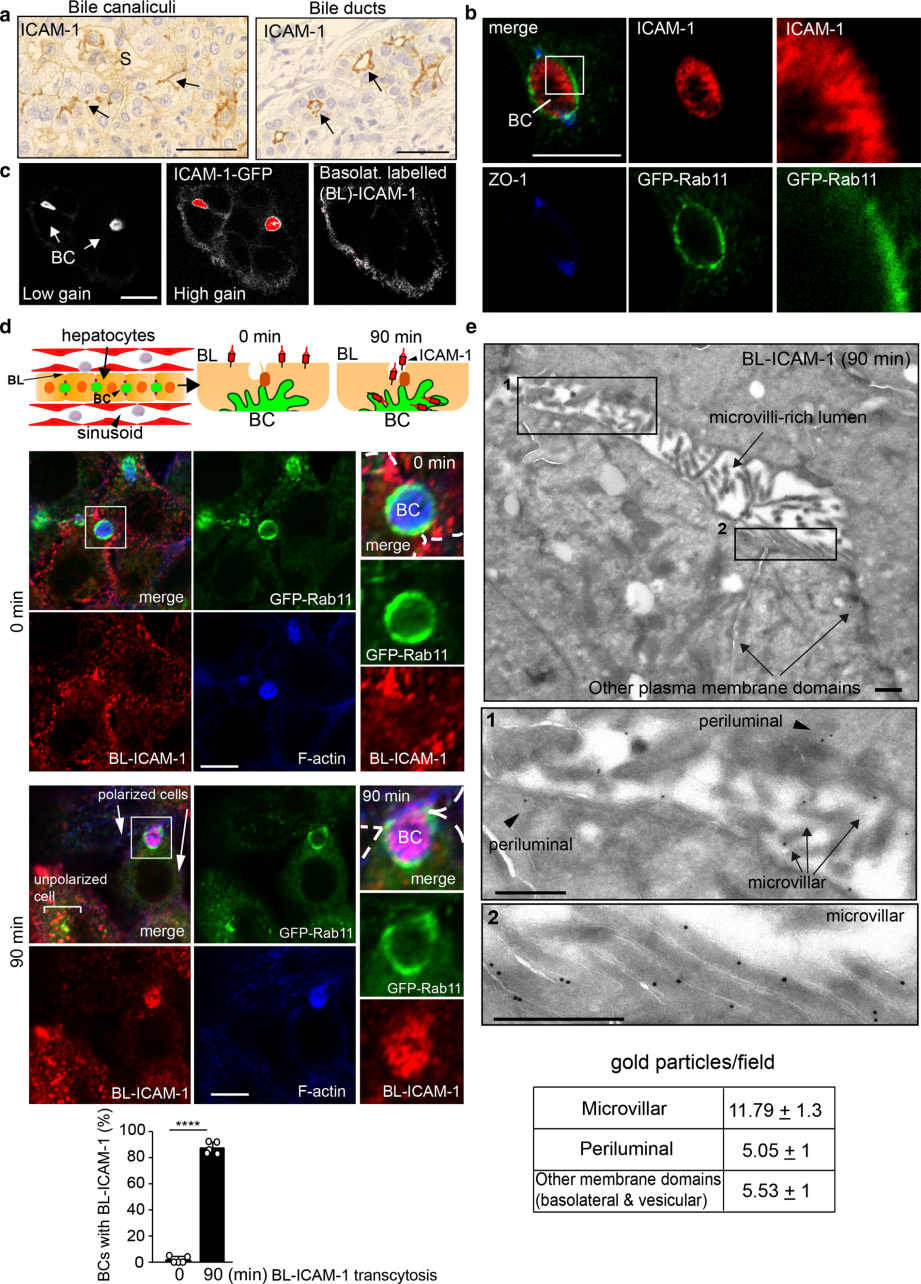
Apical localization of ICAM-1 in polarized hepatic epithelial cells is mediated by basolateral-to-apical transcytosis.** a** Immunohistochemical staining of ICAM-1 in a healthy human liver parenchyma shows receptor enrichment (arrows) in bile canaliculi (left) and bile ducts (right). (Scale bars), 50 μm. **b** HepG2 cells concentrate ICAM-1 in bile canalicular-like structures (BCs) sealed by tight junctions (TJ) and surrounded by the subapical compartment (SAC), as visualized by ZO-1 staining and GFP-Rab11, respectively. Left and central images show a single confocal plane of a BC in which the luminal and periluminal distributions of ICAM-1 and GFP-Rab11, respectively, are observed. Right images show a three-fold enlargement of the boxed area in which luminal ICAM-1 has a microvilli-like distribution. (Scale bar), 10 μm. **c** ICAM-1 can be basolaterally labeled (BL) by incubating polarized HepG2 cells with a specific antibody at 4 °C. Left and central images were taken from the same field applying different gain values in the confocal microscope, so the weak staining of the basolateral ICAM-1-GFP population could be detected with high gain settings. An antibody against ICAM-1 binds this accessible basolateral ICAM-1 population but cannot label luminal ICAM-1 (right image). Red color represents saturation of fluorescence detection in BCs. (Scale bar), 10 μm. **d** BL-ICAM-1 (arrows) translocated to BCs 90 min after increasing temperature at 37 °C. Top left cartoon illustrates the relative distribution of hepatocyte basolateral (BL) membranes and the apical bile canaliculus (BC) with respect to the sinusoids and the circulating immune cells in the liver parenchyma. Central and right cartoons illustrate the basolateral-to-apical transcytosis assay of BL-ICAM-1. Right confocal images show a three-fold enlargement of the squared areas in left images. After 90 min of transcytosis, polarized cells translocate BL-ICAM-1 to BCs whereas the receptor internalizes with a vesicular pattern and partially colocalizes with GFP-Rab11 in unpolarized cells. Single confocal planes are shown. (Scale bar), 10 μm. Bottom graph shows the quantification of BCs positive for BL-staining. *n* = 5. Between 20 and 50 BCs were quantified per experiment. Graphs show the mean ± SD. ****p < 0.0001. Discontinuous lines delineate cell borders. **e** Transmission electron microscopy revealed that translocated BL-ICAM-1 is mostly localized into microvilli-rich lumens that resemble BCs (top image). Two-fold enlargements of boxed areas in the top image are displayed below, showing the periluminal (1), microvillar (1 and 2) and vesicular, non-periluminal (3) distribution of BL-ICAM-1 after 90 min of transcytosis. Periluminal vesicles were considered those located within 1 μm distance to the microvilli-rich lumen. The table quantifies the relative distribution of BL-ICAM-1 from 19 EM micrographs. It shows the mean ± SEM. (Scale bar), 1 μm

Most of the ICAM-1 receptor is confined in apical membrane domains forming bile canalicular and ductal networks in human hepatic epithelial cells in vivo and in vitro [5] (Fig. 1a, b). HepG2 cells are spontaneously polarized human hepatic epithelial cells that form bile canalicular-like structures (BCs) with a spherical shaped-lumen sealed by tight junctions (TJs) and surrounded by the SAC, which could be detected by expressing GFP-Rab11 (Fig. 1b). These cells are thus a prototypical in vitro model for studying apicobasal polarity and intracellular trafficking [5, 15–17]. Despite its preferential apical localization, ICAM-1 also accumulates at the basolateral plasma membrane (Fig. 1c), which is accessible from the extracellular milieu. However, hepatic cells implement mechanisms to remove and translocate basolateral ICAM-1 to the BCs, where the receptor is stabilized, as we previously demonstrated by photoactivation of basolateral ICAM-1-paGFP and by antibody-mediated labelling of endogenous basolateral ICAM-1 [5]. To gain insight into the mechanisms that govern ICAM-1 polarity, we adopted the latter experimental strategy to investigate basolateral-to-apical transport of endogenous ICAM-1. The basolateral fraction of this receptor, which is exposed to sinusoids and circulating immune cells in the liver parenchyma (Fig. 1d, top), could be specifically labeled (BL-ICAM-1) with antibodies at 4 °C, which were unable to reach the main apical receptor pool in the BC (Fig. 1c). After labeling, incubation at 37 °C for 90 min reactivated membrane trafficking and basolateral-to-apical translocation of BL-ICAM-1, which could be detected by confocal microscopy (Fig. 1d, Figure S1a) [5]. It is of note that antibody-labeled ICAM-1 displayed a vesicular-like pattern at 90 min of trafficking in unpolarized cells (Fig. 1d, bottom image), suggesting that apicobasal polarity redirects the intracellular trafficking of ICAM-1. The immunolocalization of BL-ICAM-1 by transmission electron microscopy also revealed a remarkable increase of the adhesion receptor in microvilli-rich lumens that resembled BCs (Fig. 1e, Figure S1b).

**Fig. 2:**
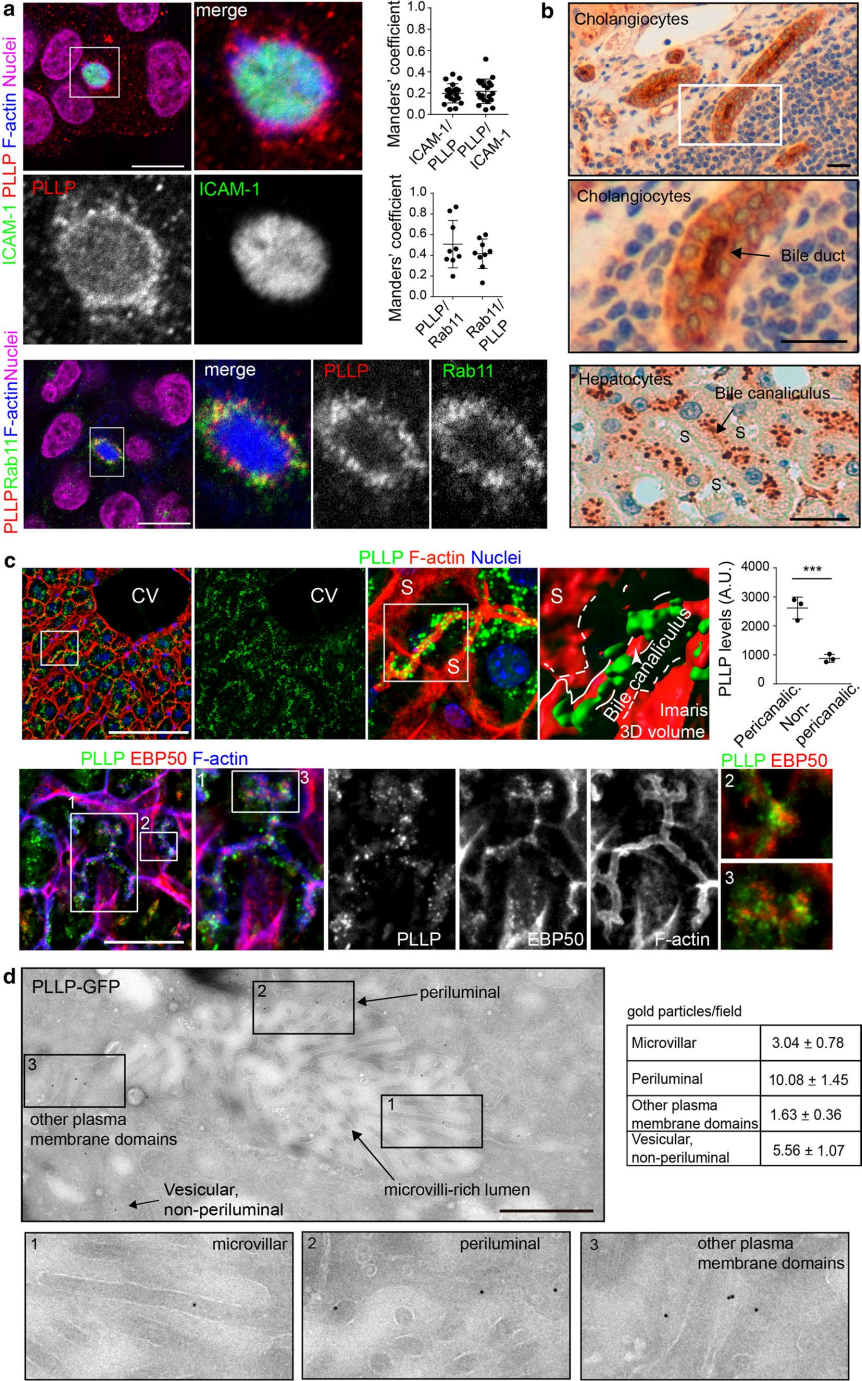
PLLP/plasmolipin is localized in the subapical compartment surrounding the bile canaliculi of polarized hepatic epithelial cells in vitro and in vivo*.* **a** Subapical distribution of endogenous PLLP. Top images. An antibody generated against hPLLP mostly stains a vesicular, periluminal compartment. Bottom images. The periluminal distribution of endogenous PLLP overlaps with that of endogenous Rab11. (Scale bars), 10 μm. The right graphs show Manders’ coefficient measurements, which expressed the fraction of pixels positive for the first protein staining which is also positive for the second protein staining. Between 20 and 9 luminal and periluminal areas were quantified in total. It shows the mean ± SD. *n* = 3. A single confocal plane is shown. **b** PLLP (arrows) is expressed in cholangiocytes (top) and hepatocytes (bottom) in livers from healthy human donors. PLLP shows an intracellular distribution in the proximity of bile canaliculi, and distal of sinusoids (S) (Scale bars), 20 μm. **c** PLLP localizes to a pericanalicular vesicular compartment in murine liver. S, sinusoids, CV, central vein. (Scale bars), 100 (top) and 20 (bottom) μm. The discontinuous lines mark sinusoids, the continuous lines mark the bile canaliculus in the Imaris 3D image. Quantification of pericanalicular vs non-pericanalicular PLLP staining. Pericanalicular area was considered that comprised within 3 μm distance to the canalicular border. *n* = 3 murine livers. Graph shows the mean ± SD. Bottom images. Bile canaliculi are enriched in F-actin and EBP50. *A.U* arbitrary units. Z-projections of 10 confocal planes of 1 μm thickness are shown **d** Immunolocalization of PLLP-GFP by transmission electron microscopy. The right table shows the quantification of PLLP-GFP distribution in different membrane structures from 36 EM images, in which the most abundant is surrounding microvilli-rich lumens that resemble BCs. Periluminal vesicles were considered those located within 1 μm distance to the microvilli-rich lumen. The table shows the mean ± SEM. (Scale bar), 1 μm

**Fig. 3:**
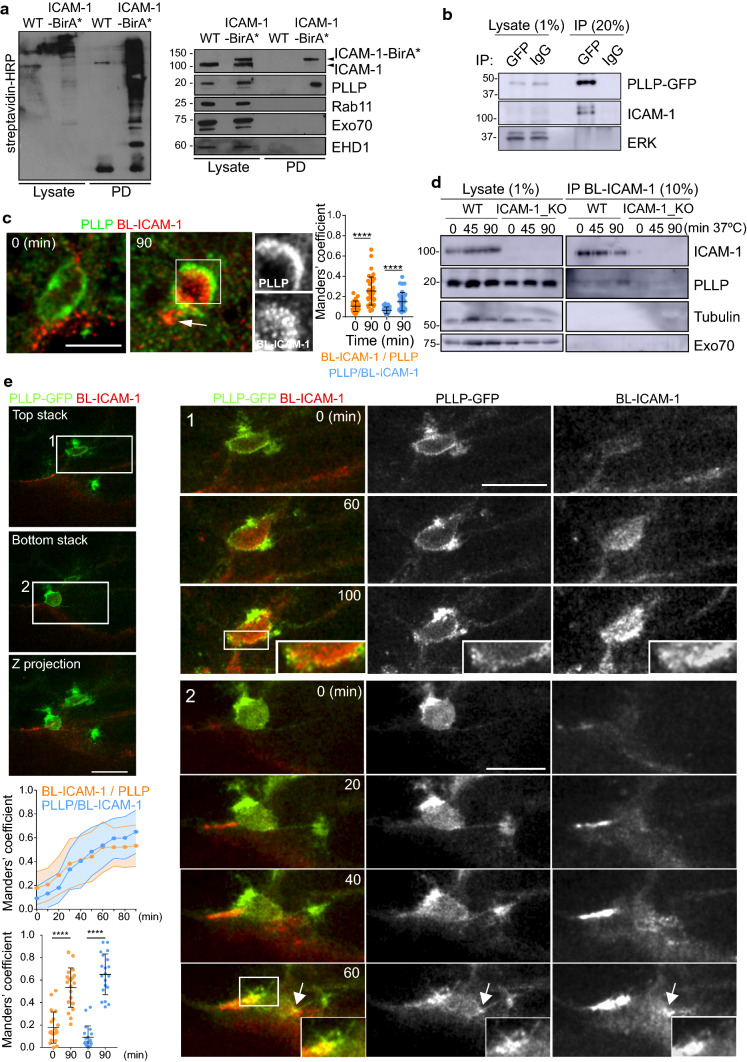
ICAM-1 associates with PLLP during the basolateral-to-apical trafficking of the receptor.** a** ICAM-1 BioID reveals the proximal interaction of the receptor with PLLP. Parental HepG2 cells and HepG2 cells stably expressing ICAM-1-BirA* were incubated with 50 μM biotin for 16 h, lysed and subjected to a pull-down (PD) assay with neutravidin-agarose. Biotinylated proteins were detected by western blot with streptavidin-HRP (left blot). Western blots show ICAM-1-BirA* biotinylation as positive controls and the specific biotinylation of PLLP but not of Rab11, Exo70 and EHD1 traffic proteins (right blots). ICAM-1 and ICAM-1-BirA* (arrowheads) were detected with anti-ICAM-1 antibody. **b** Lysates of HepG2 cells stably expressing PLLP-GFP were subjected to immunoprecipitation with rabbit anti-GFP and rabbit control IgG antibodies. ERK and tubulin blots are shown as negative controls. **c** PLLP distribution partially overlapped with that of BL-ICAM-1 after 90 min of transcytosis (arrows). ICAM-1 was basolaterally labeled (BL-ICAM-1) with a specific antibody at 4 °C in HepG2 cells (0 min). Cells were then incubated at 37 °C for 90 min. Single channels from the squared area are individually shown in greyscale on the right images. A single confocal plane is shown. (Scale bars), 5 μm. Bottom graphs show Manders’ coefficients for the distribution of the indicated pairs of proteins. *n* = 3, at least six luminal areas were quantified per experiment. **d** PLLP coimmunoprecipitates with BL-ICAM-1 during transcytosis. ICAM-1 was basolaterally labeled with antibodies in wild type (WT) HepG2 cells. Labeled cells were incubated at 37 °C for the indicated periods, lysed and the lysates were incubated with protein G-Sepharose to immunoprecipitate BL-ICAM-1 during its basolateral-to-apical traffic. The same procedure was performed in parallel with ICAM-1_KO cells as a control of the immunoprecipitation. **e** Time-lapse confocal images showing the translocation of fluorescently labeled BL-ICAM-1 to BCs in polarized cells expressing PLLP-GFP. Left images show a general view of the field of the time-lapse movies. Right images show selected frames of the two boxed areas from the left images. The insets highlight PLLP-GFP and BL-ICAM-1 overlapping regions. The arrows point at PLLP-GFP-positive tubular structures during BL-ICAM-1 translocation. (Scale bars), 10 μm. Bottom left graphs. Manders´ coefficients between PLLP-GFP and BL-ICAM-1 increase as receptor translocation progresses during the time-lapse analysis (top). Statistically significant increase of Manders’ values after 90 min of transcytosis (bottom). At least 20 BCs were quantified. The graph shows the mean ± SD. ****p < 0.0001

We searched for protein machinery that could be involved in ICAM-1 transcytosis in polarized hepatic cells. The publicly available *Human Protein Atlas* database [18–20], can be used to data-mine gene and protein expression patterns in all human tissues [19, 21]. We screened this database to identify the expression patterns of families of proteins potentially involved in transcytosis. The MAL family comprises raft-associated, integral membrane proteins containing at least one MARVEL domain, which are involved in polarized vesicular trafficking and transcytosis in various cell types [8, 9, 12]. We found high levels of mRNA coding for the MAL family member PLLP [14] in HepG2 cells and in human liver tissues (https://www.proteinatlas.org) (Figure S2). In addition, PLLP expression increases upon fibroblast reprograming into hepatocytes, suggesting a specific role for this protein in epithelial differentiation [22]. PLLP was also significantly expressed in renal, intestinal and ovarian epithelial cell lines. Other *MAL* genes, such as *MAL2* [12] and *MAL* [23] exhibited different restricted expression patterns or, like *MYADM,* were ubiquitously expressed [24] (Figure S2). We generated an anti-PLLP polyclonal antibody to the last 17 residues of the C-terminal domain of PLLP. This antibody specifically recognized a 20 kD protein PLLP, as shown by western blotting of HepG2 cells in which the *PLLP* gene was edited using CRISPR/CAS9 (PLLP_KO cells) (Figure S3a). Confocal analysis using this antibody showed periluminal staining different to the luminal distribution of ICAM-1 (Fig. 2a, top images). This periluminal distribution was not detected in PLLP_KO cells (Figure S3b). Additional colocalization analyses revealed that endogenous PLLP distribution partially overlapped in the SAC with endogenous Rab11, suggesting that these two proteins are components of the same compartment but also label different vesicular populations (Fig. 2a, bottom images). Endogenous PLLP staining was mostly confined to the SAC (Fig. 2a, bottom images) but also yielded some weak apical staining in some cells (Fig. 2a, top images), suggesting that this protein transits between these two cellular regions. In healthy human and murine hepatic tissues, PLLP was apically enriched in the bile ducts of cholangiocytes and in an intracellular vesicular pattern close to the bile canaliculi in hepatocytes (Figs. 2b, c). The antibody raised against PLLP did not yield clear results in electron microscopy experiments. Thus, we constructed an expression vector coding for PLLP-GFP and generated HepG2 cells stably expressing this fluorescent MAL protein chimera. Transmission electron microscopy of PLLP-GFP confirmed that a significant proportion of this protein is distributed in cellular regions surrounding microvilli-rich lumens resembling BCs (Fig. 2d). To investigate the potential proximal interaction between ICAM-1 and PLLP, we generated a construct of ICAM-1 conjugated from its C-terminal domain to the mutant of biotin-ligase BirA*, which adds biotin covalently to nearby proteins, and performed BioID assays in HepG2 cells that stably expressed the ICAM-1-BirA* chimera [25]. ICAM-1-BirA* expression induced the biotinylation of several proteins that could be purified by neutravidin-agarose pull–down (PD), including ICAM-1-BirA* itself (Fig. 3a). A 20 kD protein band was clearly identified in the PD fraction with the anti-PLLP antibody (Fig. 3a). In contrast, other proteins involved in polarized vesicular trafficking such as Rab11, the exocyst component Exo70 [26] and the EPS15 homology (EH) domain-containing protein EHD1 [27], were not detected in this fraction. In addition, ICAM-1 was detected in PLLP-GFP immunoprecipitations performed with anti-GFP, suggesting an association between these two proteins (Fig. 3b). Transcytosis experiments of antibody-labeled ICAM-1 (Fig. 1d) revealed that overlapping of BL-ICAM-1 staining with PLLP in subapical regions significantly increased after 90 min of trafficking (Fig. 3c). The immunoprecipitation of the antibody that labeled ICAM-1 during this trafficking assay also suggested that PLLP associates with BL-ICAM-1 in its route towards apical membrane domains (Fig. 3d). As a control, we generated *ICAM-1*-gene-edited (ICAM-1_KO) cells, in which no PLLP was detected in the immunoprecipitates (Fig. 3d). Time-lapse spinning disk confocal microscopy of BL-ICAM-1 in PLLP-GFP HepG2-cells showed an increase in the periluminal overlapping between the itinerant ICAM-1 and PLLP-GFP, as shown by Manders’ analyses of video frames (Fig. 3e). This time-lapse analysis also revealed that the PLLP compartment was highly dynamic and reorganized during ICAM-1 translocation (Fig. 3e and Video S1), transiently emitting intracellular protrusions in the 70% of BCs analyzed (Fig. 3e and Video S2). These protrusions were very similar to those described for other proteins involved in transcytosis that are enriched in intracellular membrane structures that contact basolateral cargo [16]. Double immunodetection by transmission electron microscopy confirmed the observations made in time-lapse confocal experiments and suggested that BL-ICAM-1 is translocated towards apical membrane domains by transiently or partially trafficking through PLLP–enriched vesicles (Figure S4). Interestingly, PLLP exhibited a vesicular pattern in non-polarized cells. In these cells, a proportion of these PLLP vesicles were condensed around the centrosome, similar to the distribution of proteins resident in the endosomal recycling compartment (Figure S5a, b) [28, 29]. A significant fraction of antibody-labeled ICAM-1 colocalized with PLLP in different cellular domains (Figure S5a, c), suggesting that PLLP is also part of the machinery regulating ICAM-1 trafficking in non-polarized cells, and that apicobasal polarity controls the localization of this receptor by redistributing the trafficking machinery towards the SAC. Collectively, our results suggest that ICAM-1 interacts with PLLP, an intracellular trafficking protein that is strongly expressed by hepatic epithelial cells, localized to the SAC in the proximity of BCs, where BL-ICAM-1 is preferentially transported.

### PLLP is necessary for the apical polarization of ICAM-1

**Fig. 4:**
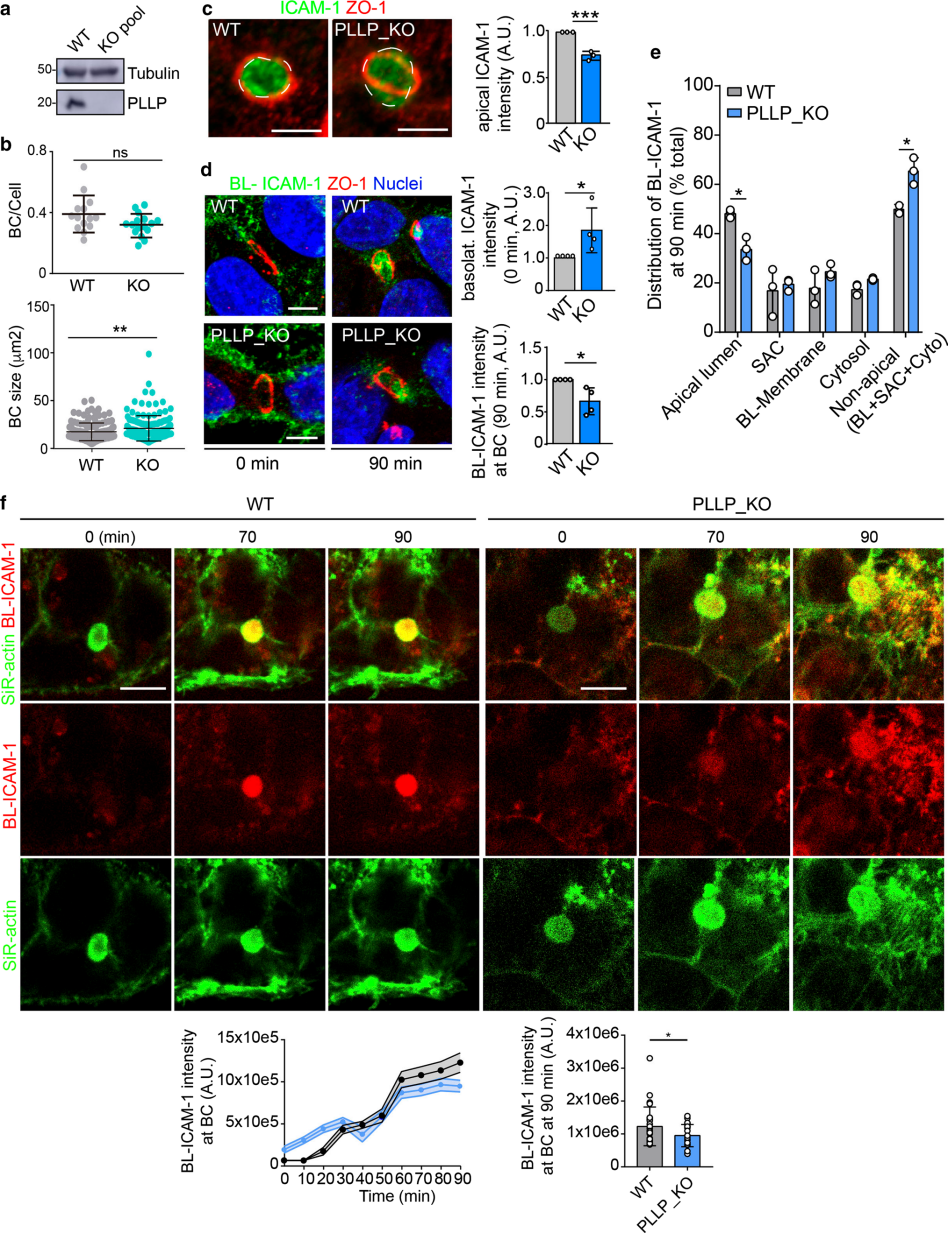
CRISPR-CAS9-mediated editing of the *PLLP* gene shows that ICAM-1 polarity is regulated by PLLP expression.** a** CRISPR-CAS9-mediated editing of the *PLLP* gene (PLLP_KO) in HepG2 cells. Several clones were selected and pooled to prevent clonal variations during the trafficking assays. WT, parental wild type cells. **b** PLLP_KO cells have similar BC frequency per cell (top graph) and slightly larger BCs (bottom graph). At least five different confocal areas per experiment were quantified. *n* = 3. **c** Relative ICAM-1 staining intensity at BCs (apical ICAM-1) in WT and PLLP_KO cells. (Scale bar), 5 μm. Discontinuous lines delineate BCs. **d** BL-ICAM-1 transcytosis to BCs (arrows) in WT and PLLP_KO cells. BL-ICAM-1 labeling intensity before transcytosis (0 min; basolat. ICAM-1) and BL-ICAM-1 staining intensity in BCs at 90 min of transcytosis (90 min; apical BL-ICAM-1) were quantified in the right graphs. (Scale bars), 5 μm. **e** Quantification of the distribution of BL-ICAM-1 at 90 min of transcytosis following the procedure described in Figure S5. The moderate increase of BL-ICAM-1 observed in each non-luminal cellular region measured on PLLP_KO cells with respect to WT cells resulted in a significant increase in overall non-apical BL-ICAM-1 in PLLP_KO cells. In accordance, a significant decrease of luminal BL-ICAM-1 was also detected in PLLP_KO cells. At least six different confocal regions were quantified per experiment. *n* = 3. *p < 0.05. **f** Time-lapse analysis of BL-ICAM-1 translocation to BCs in WT and PLLP_KO cells labeled with SiR-actin for BC visualization. Bottom left graph. Quantification of the relative fluorescence intensity of BL-ICAM-1 at BCs every ten frames. Bottom right graph. Quantification of the luminal fluorescence intensity of BL-ICAM-1 at 90 min of transcytosis. The graph shows the mean ± SD. At least 24 BCs were quantified for each cell type. *p < 0.05. (Scale bar), 10 μm. *A.U* arbitrary units

**Fig. 5:**
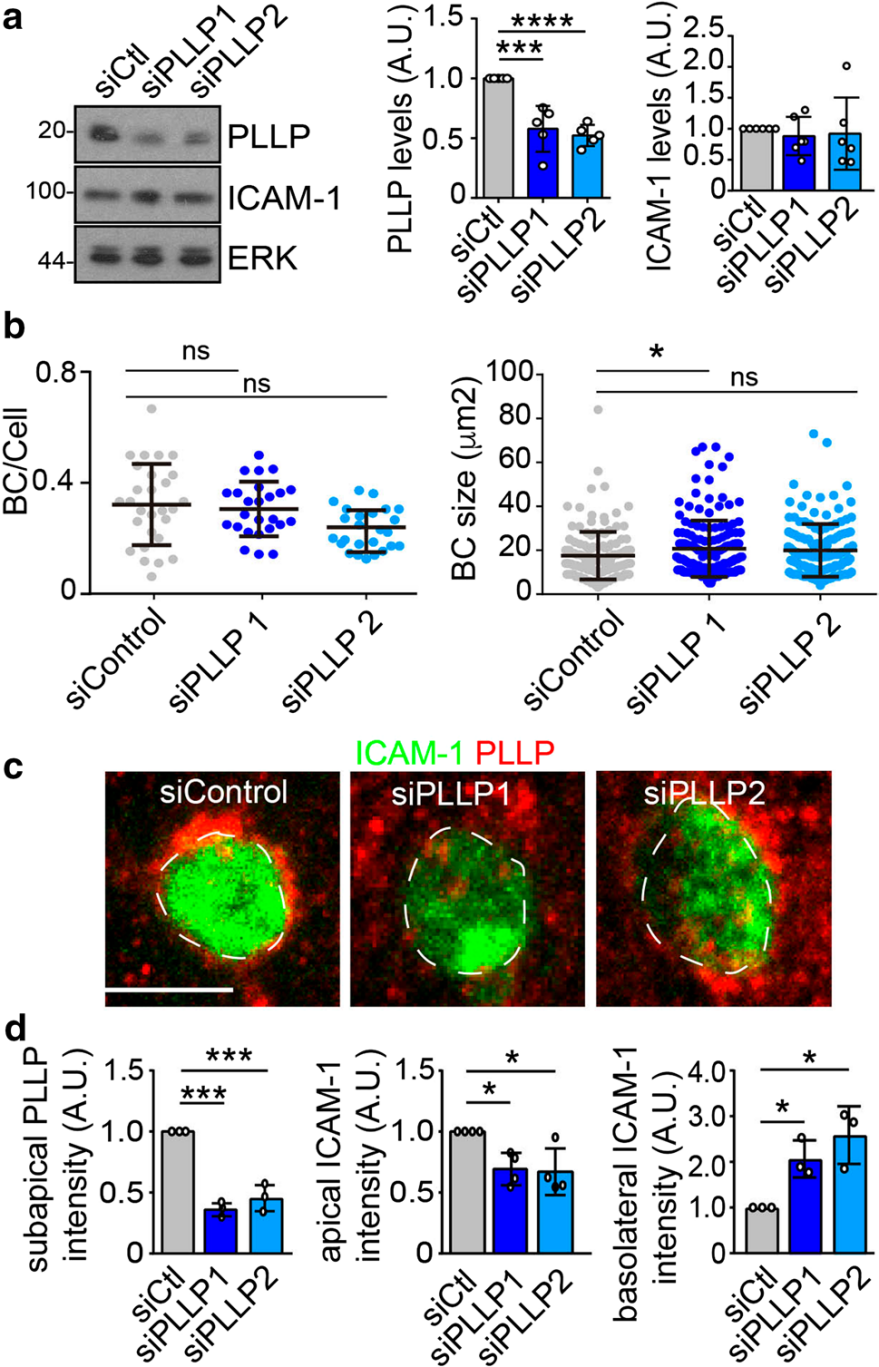
PLLP knockdown with siRNA shows that ICAM-1 polarity is regulated by PLLP expression.** a** Effect of PLLP knockdown (KD) with two siRNAs on total PLLP and ICAM-1 protein expression as determined by western blot analysis. **b** PLLP_KD cells have similar BC frequency per cell (left graph) and slightly larger BCs (right graph). At least five different coverslip fields per experiment in fourdifferent experiments were quantified **(c-d)** Effect of PLLP KD on subapical PLLP and apical and basolateral ICAM-1 staining intensities from confocal microscopy images **c**. (Scale bar), 5 μm. Discontinuous lines delineate BCs. **d**. Graphs show the mean ± SD. *p < 0.05; ***p < 0.001; ****p < 0.0001; ns, not statistically significant, *p* > 0.05. *A.U* arbitrary units

To investigate the role of PLLP in ICAM-1 trafficking, we analyzed ICAM-1 polarity and basolateral-to-apical transcytosis in parental wild type (WT) and PLLP_KO HepG2 cells. Several clones of PLLP_KO HepG2 cells were pooled to prevent clonal variations during the assays (Figures S3a, b and 4a). *PLLP* gene editing neither affected the frequency of BCs per cell (Fig. 4b, top graph) nor cellular area and perimeter (Figure S6a). However, it moderately increased cell height and BC size (Fig. 4b, bottom graph, and S6a). Absence of PLLP expression reduced the overall staining intensity of ICAM-1 in BCs (Figs. 4c and S6a), increased BL-ICAM-1 staining intensity of cells exposed to the antibody at 4 °C (Figs. 4d, top graph) and, after 90 min of trafficking, decreased basolateral-to-apical transcytosis of the receptor, measured as the staining intensity of BL-ICAM-1 in BCs (Fig. 4d, bottom graph and S6a). We further compared BL-ICAM-1 trafficking between WT and PLLP_KO cells by performing an assay of basolateral-to-apical transcytosis in which we added an additional step of surface labeling with a secondary antibody after 90 min of transcytosis and before cellular fixation (Figure S6b-d). This experiment allowed to discriminate internalized from non-internalized BL-ICAM-1 populations [30] and revealed that the absence of PLLP caused a general delay in the basolateral-to-apical trafficking of the receptor, but no specific accumulation of ICAM-1 in intracellular compartments such as vesicles or the SAC, the latter detected by expressing GFP-Rab11 (Fig. 4e and S6d). This delay was similar to that found in previous measurements (Fig. 4d, bottom graph) and was also observed in the time-lapse spinning disk confocal microscopy assays, in which BCs were labeled by incubating cells with the fluorescent probe SiR-actin during the transcytosis assay (Fig. 4f, Videos S3, S4). This trafficking delay observed at 90 min of transcytosis, may therefore contribute to the basolateral increase of almost 100% and the apical decrease of 25% observed for total ICAM-1 distribution (Fig. 4c and 4d, top graph). It is of note that these defects in ICAM-1 transcytosis in PLLP_KO cells may also involve diverting the receptor to alternative trafficking routes worth exploring in the future, such as basolateral receptor recycling. Next, *PLLP* gene expression was silenced with two siRNAs, which caused a 50% decrease in PLLP protein levels by immunoblot (Fig. 5a). Similar to PLLP_KO cells, PLLP_KD cells had BC frequency similar to that observed in control cells and a tendency to increase BC size (Fig. 5b). PLLP-KD cells had a 60% decrease in PLLP staining in the subapical compartment (Fig. 5c, d, left graph). ICAM-1 protein expression was not altered upon PLLP knockdown (Fig. 5a, right graph) but luminal staining intensity in BCs decreased (Fig. 5d, central graph) and basolateral ICAM-1 levels increased (Fig. 5d, right graph). These findings are consistent with those of experiments with PLLP_KO cells and, collectively, indicate that PLLP specifically participates in the basolateral ICAM-1 sorting to the apical plasma membrane domain.

### PLLP regulates ICAM-1-dependent adhesion of T-lymphocytes to polarized hepatic epithelial cells

**Fig. 6:**
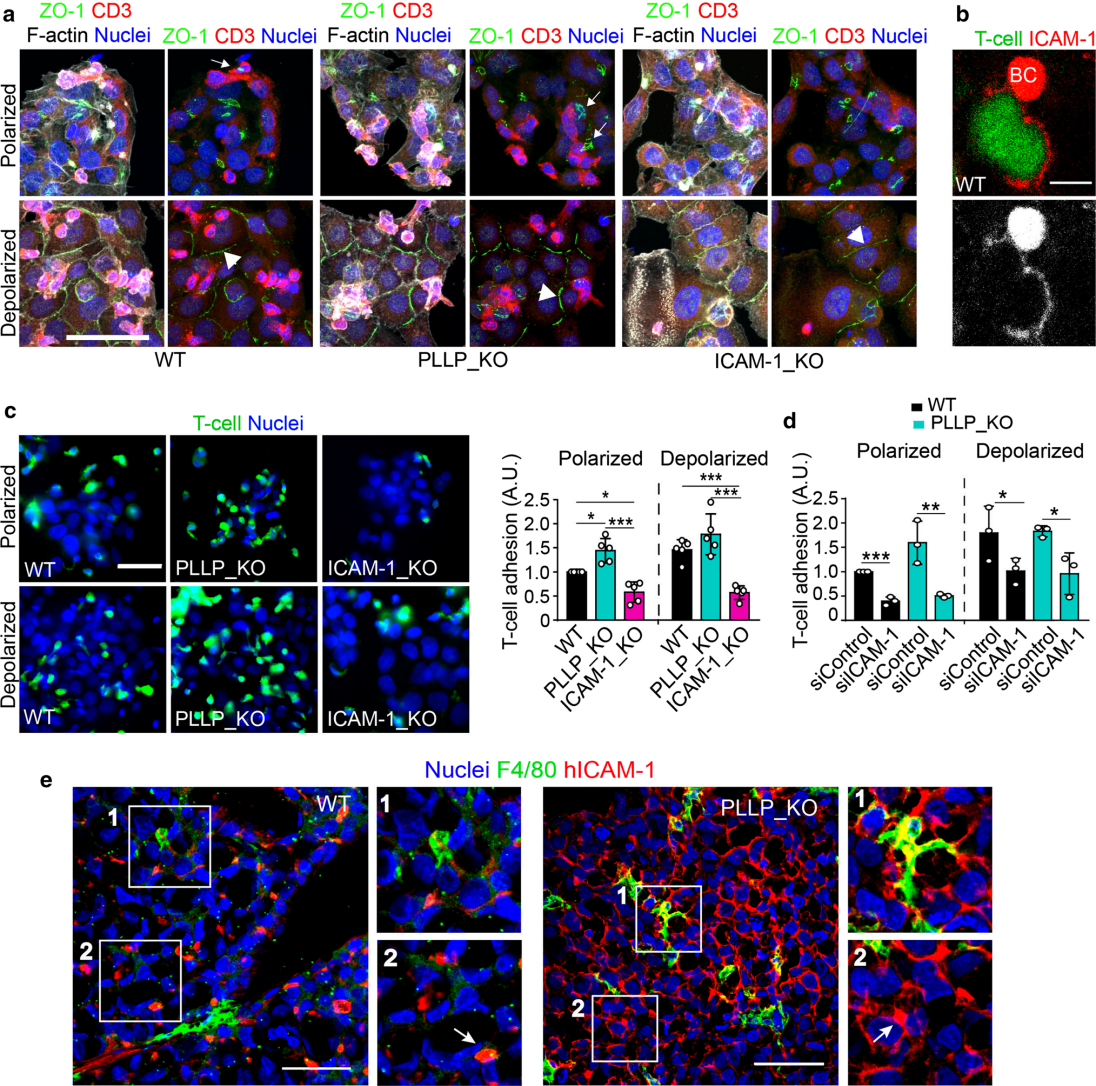
PLLP regulates T-lymphocyte adhesion to polarized hepatic cells. **a** Top images. Adhesion of human memory T-lymphocytes (T cells) to hepatic epithelial cells for 15 min at 37 °C did not induce BC depolarization. Arrows point at BCs, detected by F-actin and ZO-1 double staining, in the proximity of T cells, detected with by CD3 staining. Bottom images. Compared to cells in the top images, F-actin is not forming BCs and ZO-1 redistributes to linear cell–cell junctions (arrowheads) upon the depolarizing treatment with PMA for 2 h and the subsequent extensively washings, carried out prior the adhesion assay. (Scale bar), 100 μm. Z-projections of at least nine confocal planes of 0.8 μm thickness are shown **b** Representative image of a calcein-labeled T cell (green) adhered to a hepatic epithelial cell and surrounded by ICAM-1-rich membrane protrusions. The top red cluster corresponds to a BC. **c,d** Increased T-lymphocyte adhesion to PLLP_KO hepatic epithelial cells. Control wild type (WT), PLLP_KO and ICAM-1_KO HepG2 cells were incubated with calcein-labeled T cells for 15 min at 37 °C. The T-cell adhesion increase detected in PLLP_KO cells was similar to that observed when cells were previously subjected to the depolarizing treatment. Polarized and depolarized ICAM-1_KO cells exhibited remarkably reduced adhesion to T cells. *n* = 5. **b** shows representative images taken with a florescence microscope of the experiments quantified in (**c**). (Scale bars), 5 (right), and 100 (left) μm. *n* = 5. **d** The T-cell adhesion increase in PLLP_KO relative to control cells depends on ICAM-1 expression and apicobasal polarity. WT and PLLP_KO cells were transfected with the indicated siRNA oligonucleotides. 72 h post-transfection, adhesion assays with calcein-labeled T-lymphocytes were performed and quantified. *n* = 3. **e** HepG2 cells were subcutaneously injected into nude mice to induce tumors. Mice were sacrificed 8 weeks later. Tumors were fixed and analyzed by confocal microscopy. Macrophage infiltration was detected with an anti F4/80 antibody (boxed area 1). BC-like ICAM-1 staining (arrows in boxed area 2) in HepG2 cells was analyzed with an antibody specific for human ICAM-1 (hICAM-1). Note that ICAM-1 is less polarized in PLLP_KO cells. Z-projections of at least four confocal planes of 1.4 μm thickness are shown. Graphs show the mean ± SD. *p < 0.05; **p < 0.01; ***p < 0.001. *A.U* arbitrary units

Apicobasal polarity of hepatic epithelial cells regulates ICAM-1-dependent T lymphocyte adhesion [5]. Hepatic ICAM-1 exposed to the extracellular milieu engulfs T cells and mediates their firm adhesion to HepG2 cells [31]. To test whether the partial depolarization of ICAM-1 affects leukocyte adhesion to hepatic cells depleted in PLLP, we performed adhesion experiments using memory T-lymphocytes from human blood (T cells) as previously described [5] (Fig. 6a–c). We exposed epithelial cells to T cells for no longer than 15 min to prevent potential T cell-induced hepatic cell depolarization (Fig. 6a, top). Parallel experiments with epithelial cells subjected to a previous depolarization treatment [5] were carried out to investigate the contribution of apicobasal polarity to T-cell adhesion (Fig. 6a, bottom). Adhesion assays were performed with calcein-labeled T lymphocytes to better quantify the fluorescence of adhered cells (Fig. 6b, c). *PLLP* gene-edited (PLLP_KO) cells exhibited greater T-cell adhesion compared with WT cells (Fig. 6a, c). In contrast, *ICAM-1*-gene-edited (ICAM-1_KO) cells had a lower capacity to adhere T-cells, confirming the central role of ICAM-1 in epithelial-lymphocyte interaction [5]. It is of note that differences in T-cell adhesion between WT and PLLP_KO cells were attenuated when cells were depolarized before conducting the adhesion assay (Fig. 6a, c) showing the importance in these adhesion assays of apicobasal polarity, which determines ICAM-1 localization. To confirm that the effect of PLLP_KO was dependent on ICAM-1 expression, ICAM-1 was silenced in WT and PLLP_KO cells, and T-cell adhesion assays were performed (Fig. 6d). The adhesion increase caused by *PLLP* knockout was reversed when ICAM-1 was depleted, indicating that such an increase was dependent on ICAM-1 expression. Finally, tumors were induced by subcutaneous injection of HepG2 cells and analyzed for ICAM-1 distribution and macrophage infiltration. Quantification of tumor size progression suggested that wild type and PLLP_KO tumors had similar growth rates (Figure S7a). Macrophage infiltration was more frequently detected in PLLP_KO tumors than in control tumors although a remarkable degree of variation was observed between samples (Figure S7b). Importantly, ICAM-1 from HepG2 cells colocalized with cells positive for the macrophage marker F4/80 (Fig. 6e, boxed area 1), suggesting that hepatoma cells interact with immune cells infiltrated in the tumor. In addition, ICAM-1 was concentrated in structures resembling BCs in tumors from WT cells, whereas its distribution was less polarized in tumors from PLLP_KO cells (Fig. 6e, boxed area 2), which is consistent with the results obtained from the experiments performed in vitro (Fig. 4).

## Discussion

The polarized architecture of epithelial cells has recently emerged as a mechanism for modulating the intensity of the inflammatory response. In intestinal epithelial cells, the apical polarization of ICAM-1 establishes a haptotactic gradient and a directional migration of leukocytes across the epithelial barrier [2]. ICAM-1 polarization also allows immune cells to discriminate between polarized and depolarized hepatic epithelial cells [5]. Hence, the sorting machinery involved in the surface localization of adhesion receptors could be a previously unknown regulatory element for the adhesion and migration of lymphocytes on polarized epithelia. PLLP belongs to the MAL family, which is composed of integral membrane proteins that are highly hydrophobic and contain at least one MARVEL domain [9]. Most of them can be isolated with organic solvents used to extract lipids, so they are called proteolipids [32]. MAL proteins contain short C- and N-terminal segments facing the cytoplasmic side of the leaflet, although their functional interactions with other proteins are largely determined by their compatibility with liquid–ordered plasma membrane domains or lipid rafts, which are, indeed, organized by this family of proteins [10, 33]. The relevance of intracellular trafficking machinery and, in particular, of MAL family proteins in modulating immune and inflammatory responses had been previously demonstrated in other cellular contexts. For example, the founding member of the family, MAL, regulates the transport to the immunological synapse of essential signaling components in T cells, such as the src kinase Lck, which is palmitoylated and also resides in liquid-ordered domains [34]. MYADM, another MAL protein that is almost ubiquitously expressed, negatively regulates ezrin-radixin-moesin (ERM) proteins and the actin cytoskeleton at endothelial cell–cell junctions. Its depletion reduces endothelial barrier function and induces a mild inflammatory status that increases leukocyte adhesion through ICAM-1 expression [24]. Although MYADM resides in the plasma membrane and does not participate in endothelial ICAM-1 trafficking, its functional relationship with ICAM-1 is an evidence on the compatibility of this transmembrane receptor with liquid-ordered membrane domains. It has been previously reported that ICAM-1 engagement segregates this receptor in raft-like membrane domains, which regulates its signaling abilities and reduces the association of ICAM-1 with src kinases [30, 35, 36]. It is thus reasonable that the machinery interacting and transporting ICAM-1 to specific cell surface domains participates in the organization of these plasma membrane domains [10].

Intracellular ICAM-1 trafficking has not been addressed in detail other than as a receptor for functional nanocarriers for drug delivery [37] or as a receptor for rhinovirus [38], despite the receptor relevance in the inflammatory response, and no proximal interaction of ICAM-1 with polarized intracellular trafficking machinery had previously been reported. In TNF-stimulated endothelial cells, ICAM-1 undergoes apical-to-basolateral transcytosis through caveola upon engagement, which regulates leukocyte diapedesis [30]. Interestingly, HepG2 cells do not contain caveola and express very low levels of the main constituent of caveolae, caveolin-1. PLLP and caveolin-1 share functional features such as being organizers of liquid-ordered plasma membrane domains. Indeed, ectopic expression of caveolin-1 in HepG2 revealed very significant colocalization with engaged ICAM-1 (not shown), suggesting that caveolae-rich epithelial cells, such as regenerating hepatocytes, may also transport ICAM-1 via this system of intracellular vesicles. At least one other MAL protein, MAL2, is also expressed in polarized hepatic epithelial cells. Our findings indicate that PLLP selectively regulates the polarity of ICAM-1 but not the whole apicobasal polarity in HepG2. In contrast, MAL2 depletion affects transcytosis and apicobasal polarization of hepatic and non-hepatic epithelial cells [12, 16]. MAL2 and PLLP reside in the subapical compartment and their functional relationship is currently unknown, but it would be of great interest to address whether these two proteins play additive functions in the polarized intracellular transport machinery. Likewise, PLLP and Rab11 reside in the SAC but do not perfectly colocalize. The functional relationship between Rab11 and these MAL proteins, as well as the contribution to PLLP function of other subapical Rabs potentially involved in the SAC-mediated intracellular trafficking [39], have yet to be investigated.

Inflammatory signaling induces a remarkable transcriptional program that makes it possible to synthesize adhesion surface receptors as well as soluble factors, such as cytokines and chemokines that signal to and interact with circulating immune cells. This program thereby transiently exposes the intracellular trafficking machinery to a temporary stress which may turn the expression of its components into a rate-limiting step in the inflammatory response [40, 41]. Cytokine secretion is tightly regulated by SNARE and SNARE-associated proteins [41, 42] as is the secretion of cytolytic granules in leukocytes [43] and of Weibel-Palade bodies in the endothelium during the earliest stages of the inflammatory response [44]. Transcytosis, in particular, plays an important role in the immune response because it is required for antigen and immunoglobulin transport in polarized epithelia. The polymeric Ig receptor (PigR) mediates the basolateral-to-apical transcytosis of IgA, which is essential for mucosal immunological response [45]. Here, we show that the SNARE-associated protein PLLP is an essential component of the machinery controlling the transcytosis and the surface localization of ICAM-1, an essential adhesion receptor in epithelial cells. This highlights the importance of the intracellular trafficking machinery during the immune and inflammatory response and identifies new molecular targets of biomedical interest that modulate these responses.

## Star methods

### Key resources table

**Table:** 

Reagent or resource	Source	Identifier
Mouse anti-ICAM-1	R&D Systems	#BBA3; IF 1/400; IP 1/100; RRID: AB_356950
Rabbit anti-ICAM-1	Santa Cruz Biotechnology	sc-7891; WB 1/1000; RRID: AB_647486
Mouse anti-ICAM-1	Santa Cruz Biotechnology	sc-107; IHC 1/1000; RRID: AB_627120
Rat anti-ICAM-1	EBioscience	14–0542-81; WB 1/1000; IF 1/200; RRID: AB_529544
Rabbit anti-PLLP	Our laboratory	WB 1/1000; IF 1/400; IHC 1/1000; IP 1/100
Mouse anti-PLLP	Our laboratory	IF 1/400
Rabbit anti-ERK1/2	Santa Cruz Biotechnology	sc-94; WB 1/1000; AB_2140110
Mouse anti-tubulin	Santa Cruz Biotechnology	sc-134241; WB 1/5000; RRID: AB_2009282
Rabbit anti-γ-tubulin	Sigma Aldrich	T3559; IF 1/2000
Mouse anti-CD3 OKT3	ATCC	IF 1/50 from supernatant generated from mouse hybridoma
Mouse anti-GFP	Roche	11,814,460,001; WB 1/1000; RRID: AB_390913
Rabbit anti-ZO-1	Thermo Fisher Scientific	40–2200; IF 1/500; RRID: AB_2533456
Rabbit anti-Rab11	Thermo Fisher Scientific	71–5300; WB 1/1000; IF 1/100 RRID: AB_2533987
Mouse anti-Exo70	MERCK (Millipore)	MABT186 clone 70X13F3; WB 1/500
Mouse anti-IgG	MERCK (Sigma-Aldrich)	I5381; IP 1/100; RRID: AB_1163670
Rabbit anti-IgG	MERCK (Sigma-Aldrich)	I5006; IP 1/100; RRID: AB_1163659
Rabbit anti-EHD1	MERCK (Sigma-Aldrich)	SAB2105824; WB 1/1000; RRID: AB_10740603
Rat anti-F4/80	Abcam	ab6640; IF 1/1000; RRID: AB_1140040
Goat F(ab) anti mouse IgG 596	Abcam	Abcam (ab6723); IF 1/100; RRID: AB_955573
Streptavidin-HRP	Thermo Fisher Scientific	815–968-0747; WB 1/10000
Phalloidin-Alexa Fluor 647	Thermo Fisher Scientific	A-22287; IF 1/250; RRID:AB_2620155
SiR-actin	Spirochrome	TimeLapse 1/5000
DAPI	MERCK	268,298; IF 1/1000
Donkey anti-mouse Alexa Fluor 488	Thermo Fisher Scientific	A-21202; IF 1/500; RRID: AB_141607
Donkey anti-mouse Alexa Fluor 555	Thermo Fisher Scientific	A-31570; IF 1/500; RRID: AB_2536180
Donkey anti-mouse Alexa Fluor 647	Thermo Fisher Scientific	A-31571; IF 1/500; RRID: AB_162542
Donkey anti-rabbit Alexa Fluor 488	Thermo Fisher Scientific	A-21206; IF 1/500; RRID: AB_141708
Donkey anti-rabbit Alexa Fluor 555	Thermo Fisher Scientific	A-31572; IF 1/500; RRID: AB_162543
Donkey anti-rabbit Alexa Fluor 647	Thermo Fisher Scientific	A-31573; IF 1/500; RRID: AB_2536183
Donkey anti-rat Alexa Fluor 488	Thermo Fisher Scientific	A-21208; IF 1/500; RRID: AB_2535794
Donkey anti-mouse HRP	Jackson Immunoresearch	715–035-151; WB 1/5000; RRID: AB_2340771
Donkey anti-rabbit HRP	GE Healthcare	NA934; WB 1/5000; RRID: AB_772206

### Chemicals

**Table:** 

Reagent or resource	Source	Identifier
Geneticin	Santa Cruz Biotechnology	29065B
Ultrapure salmon sperm DNA solution	Thermo Fisher Scientific	15,632
Neutravidin Agarose	Thermo Fisher Scientific	29,201
Glutathione Sepharose	GE Healthcare	17–0756-01
Phorbol 12-miristate 13-acetate (PMA)	MERCK (Sigma-Aldrich)	P8139
Ficoll	STEMCELL Technologies	07,801
PHA	Thermo Fisher Scientific	10,576,015
IL-2	Thermo Fisher Scientific	PHC0021
Calcein-AM	Thermo Fisher Scientific	C3099
Biotin	MERCK (Sigma Aldrich)	B4501
Tissue-Tek^®^ O.C.T.^™^	Sakura	4583

### Plasmids

**Table:** 

Plasmid	Backbone	Origin
ICAM-1-BirA*	pEGFP-N1	This paper
ICAM-1-GFP	pEGFP-N1	Dr. F. Sánchez-Madrid (Madrid, Spain) (Barreiro et al., 2002)
pSpCas9(BB)-2ª-GFP	PX458	Addgene; 48,138; Dr F. Zhang (Cambridge, MA)
GFP	pEGFP-N1	Clontech
GFP-Rab11	pEGFP-C1	Dr. F. Martín-Belmonte (Madrid, Spain) (Rodriguez-Fraticelli et al., 2015)
PLLP-GFP	pEGFP-N1	This paper

### sgRNAs CRISPR/Cas9

**Table:** 

sgRNA name	5'–3' Sequence
sgRNA ICAM-1 fw	CACCGCGCACTCCTGGTCCTGCTCG
sgRNA ICAM-1 rv	AAACCGAGCAGGACCAGGAGTGCGC
sgRNA PLLP fw	CACCGGTCCGCGTGCTAACTTTCGA
sgRNA PLLP rv	AAACTCGAAAGTTAGCACGCGGACC

### Cloning oligonucleotides

**Table:** 

Oligonucleotide name	5'–3' Sequence
(PLLP-GFP) Plas1. 5GFPN	GGGCCCCTCGAGATGGCCGAGTTCCCGTCGAAAGTTAGC
(PLLP-GFP) Plas1. 3GFPN	CCCGGGGAATTCGGCATAGCCGCCAGCCATCTG

### siRNA oligonucleotides

**Table:** 

siRNA name	5'–3' Sequence/Target sequence (TS)
siControl	AUGUAUUGGCCUGUAUUAGUU
siICAM-1 3’UTR	GAACAGAGUGGAAGACAUAUU
siPLLP 01	(TS) GAGUUCCCGUCGAAAGUUA
siPLLP 2 (3’UTR)	GUAUAAGCCUAACAAGCAAUU

### Lead contact and materials availability

Further information and requests for resources and reagents should be directed to and will be fulfilled by the Lead Contact, Jaime Millán (jmillan@cbm.csic.es).

All unique/stable reagents generated in this study will be made available on request, but we may require a Materials Transfer Agreement.

## Methods

### Cells and culture

Human polarized hepatic HepG2 cells (70,000 cells/cm^2^) were grown at 37°C under a humidified 95% air and 5% CO_2_ atmosphere in high-glucose Dulbecco’s modified Eagle’s medium supplemented with 5% fetal bovine serum. T-lymphoblasts were prepared from isolated human peripheral blood mononuclear cells (PBMCs). Nonadherent PBMCs were stimulated with 0.5% phytohemagglutinin for 48 h and maintained in a RPMI medium supplemented with 2 U/ml IL-2 as previously described [36]. These memory T-lymphocytes were used in experiments after they had been cultured for 7–12 days.

### Cell transfection and stable expression of exogenous proteins

5 μg DNA/10^6^ cells or 100 nM siRNA/10^6^ cells were transfected by electroporation (200 mV, 950 μF and 480 Ω; Bio-Rad). Expression analysis was performed 24–48 h post-transfection, and the siRNA effect was analyzed 72 h post-transfection. For stable expression of exogenous proteins, transfected cells were selected by treatment with 0.75 μg/ml G-418 sulfate for at least 4 weeks after transfection. Positive cell clones were selected and maintained in drug-free medium. After several passages in this medium, > 80% of cells retained expression of the exogenous protein. For CRISPR/Cas9 gene editing, the cDNA sequence was analyzed using the Breaking-Cas tool (http://bioinfogp.cnb.csic.es/tools/breakingcas), and the selected target sequences were inserted in the pSpCas9(BB)-2A-GFP plasmid, which was a gift from Feng Zhang (Massachusetts Institute of Technology, Cambridge, MA, USA) (Addgene plasmid # 48,138; http://n2t.net/addgene:48138; RRID:Addgene_48138) [46]. GFP-positive cells were sorted after 24 h of transfection and plated. Individual clones were tested by immunofluorescence and immunoblot analyses.

### Generation of anti-human PLLP polyclonal antibody

The last 17 residues from the C-terminal end of PLLP were synthesized in the peptide synthesis facility of the CBM Severo Ochoa and conjugated to KLH. Two New Zealand white rabbits were immunized with 250 μg of the KLH-peptide and 30 days later the animals were subjected to three additional boosts of 125 μg, keeping 30-day intervals between boosts. Specificity of the sera was tested by western blot of lysates from cells expressing exogenous human PLLP and cells lacking PLLP by CRISPR/CAS9-mediated editing of the gene. Immunization and boosts were carried out in Vivotecnia (Tres Cantos, Spain).

### Immunofluorescence analysis by confocal microscopy

HepG2 cells were grown on coverslips, fixed in 4% paraformaldehyde (PFA) for 15 min, rinsed and treated with 10 mM glycine for 2 min to quench the aldehyde groups. Immunostaining was performed as described [47]. Briefly, cells were then permeabilized with 0.2% Triton X-100, rinsed and blocked with 3% bovine serum albumin (BSA) in PBS for 15 min at room temperature (RT). Cells were incubated for 30 min with the primary antibodies, rinsed in PBS and incubated for 30 min with the appropriate fluorescent secondary antibodies. Actin filaments were detected with fluorophore-conjugated phalloidin (see table). Incubation with antibodies and other fluorescence reagents were always performed at 37 °C. Confocal laser scanning microscopy was carried out using a confocal Zeiss LSM710 system coupled to AxioImager M2 microscope, a confocal Zeiss LSM 800 system coupled to an AxioObserver microscope, and a confocal Nikon AR1 + system coupled to an Eclipse Ti-E microscope. For time-lapse movies of PLLP-GFP HepG2 cells a confocal Spinning disk SpiSR10 microscope. Fixed cells were imaged on glass cover slips mounted in Fluoromount. Images were captured with 1024 × 1024 µm of resolution. The basolateral and apical intensities of ICAM-1 of WT and PLLP_KO HepG2 cells were calculated from confocal images of polarized cell colonies by measuring the fluorescence intensity of basolateral and apical areas, respectively. Images were processed with Fiji software and Imaris software for 3D volume reconstruction. When specified, XZ projections, which result from the sums of the confocal images containing the structure of interest, were shown.

### Basolateral ICAM-1 labeling and transcytosis

Basolaterally labeled (BL)-ICAM-1 traffic was analyzed by incubating cells with anti-ICAM-1 antibody (0.5 µg/ml) for 30 min at 4 °C. Cells were rinsed and incubated at 37 °C for the indicated times to follow the ICAM-1 basolateral-to-apical transcytotic transport. After fixation and permeabilization, antibody-labeled receptor distribution was detected by immunofluorescence with an appropriate fluorescent secondary antibody. To identify regions of ICAM-1 internalization, a BL-ICAM-1 transcytosis assay was performed for 90 min and, before fixation, cells were incubated at 4 °C in the presence of a FITC-conjugated secondary antibody. This first secondary antibody stained the surface BL-ICAM-1 population that had not yet been internalized. Cells were rinsed, fixed and permeabilized. An incubation with a second secondary antibody conjugated to a different fluorophore was carried out to identify the BL-ICAM-1 population that could not be accessed by the FITC-conjugated secondary antibody during the first incubation. This population mostly corresponded to the internalized and transcytosed fractions of BL-ICAM-1 receptor, although a minor population of surface receptor that was not bound to the first secondary antibody, was also stained in this second incubation. Time–lapse confocal microscopy of the BL-ICAM-1 transcytosis assay was performed in HepG2 cells stably expressing GFP-ZO-1 or PLLP-GFP. Briefly, cells were seeded onto eight-well culture chambers (Ibidi) and cultured for 3 days. Antibody basolateral labeling of ICAM-1 was performed at 4 °C, cells were rinsed and incubated at 4 °C for 30 min with a TexasRed-conjugated F (ab) antibody. Cells were rinsed and placed in the humidified chamber (5% CO_2_ at 37 °C) of a Nikon AR1 confocal microscope. Time-lapse was performed using a 63×/1.2 water objective lens; cells were imaged in phenol-red-free DMEM medium, buffered with 10 mM HEPES, pH 7.4, at 5–10-min intervals, depending on the experiment, for the indicated times. Time-lapse acquisitions were processed using the Fiji image processing software.

### T-lymphocyte adhesion assays

To measure the ability of HepG2 cells to adhere to T cells, HepG2 cells were plated onto 24-well plates (50×10^3^/well) for 48 h. T-lymphoblasts were labeled with 0.5 μM calcein-AM for 30 min, and extensively rinsed with medium. HepG2 cells were exposed or not to 100 nM PMA for 2 h to induce depolarization and then extensively washed before performing the adhesion assay [5]. HepG2 cells were co-cultured with T-lymphocytes in a 2:1 ratio for 15 min. After washing, cells were fixed, immunofluorescence was performed and the percentage of calcein-labeled T-lymphocytes or CD3-stained T cells adhered to HepG2 cells was measured using a fluorescence microscope.

### Tissue immunofluorescence and immunohistochemistry

The livers of the mice were removed and fixed overnight in 10% neutral buffered formalin (Sigma-Aldrich) at RT. After fixation, tissues were incubated in 30% sucrose overnight and then frozen in Tissue-Tek O.C.T. compound. The sections were allowed to cool at RT and then incubated in blocking buffer (1% DMSO, 2% BSA and 0.3% Triton X-100 in PBS) for 2 h at RT. Primary antibodies were diluted in blocking buffer and incubated overnight at 4 °C. Secondary antibodies were diluted in PBS containing 0.05% BSA and incubated for 2 h at RT. Quantification of the pericanalicular vs non-pericanalicular distribution of PLLP was performed by establishing in Fiji a ROI comprising the perimeter of the BC and then widening such perimeter by 3 μm. The PLLP staining intensity in this enlarged ROI was considered to be pericanalicular and was subtracted from the intensity in the whole cellular area, which resulted in the values of non-pericanalicular intensity.

The immunohistochemical analysis of human hepatic tissue was approved by the Hospital Ethics Committee of the Hospital Universitario de Salamanca. Biopsies from healthy donors were analysed. Formalin-fixed, paraffin-embedded sections of 4 µm thickness were deparaffinized in xylene and rehydrated through a decreasing graded ethanol solution series. After suppression of endogenous peroxidase activity (3% hydrogen peroxide, 10 min) and antigen retrieval (boiling in 10 mM citrate buffer, pH 6.0), immunostaining was performed with the appropriate primary antibody. Immunohistochemical techniques were performed with an automated Leica Bond III® system. The stained protein was visualized using DAB solution (Dako), and lightly counterstained with Mayers–haematoxylin (Leica®). To ascertain the specificity of the antibody immunoreactivity, a negative control was carried out in the absence of the primary antibody. In this case, no immunolabeling was detected.

Immunolocalization of BL-ICAM-1 and PLLP-GFP by transmission electron microscopy. For transmission electron microscopy analysis, HepG2 cells were grown on 24-well plates for 48–72 h and basolateral labeling of ICAM-1 was performed as described. After 90 min of temperature shift at 37 °C, cells were fixed in 4% PFA and 0.05% glutaraldehyde (GLA) in 0.1 M PHEM buffer, pH 6.9, for 2 h at RT and 16 h at 4 °C. After extensive washing, cells samples were embedded in 10% gelatin. Sample blocks (< 1 mm^3^) were cryoprotected with 2.3 M sucrose at 4 °C for 16 h and rapidly frozen by immersion in liquid nitrogen. Samples were sectioned on an EM FCS cryo-ultramicrotome (Ultracut UCT, Leica) at −120 °C and collected in a mixture of 2.3 M sucrose and 2% methylcellulose solution (vol/vol 1:1). Immunogold labeling was essentially performed as previously described [48]. For labeling of BL- ICAM-1 at 0 and 90-min time points, thawed 90-nm-thick cryosections were incubated for 30 min at RT with rabbit anti-mouse antibody (Dako, Denmark) followed by protein A conjugated to 15 nm gold (Cell Microscopy Core (CMC), Utrecht, The Netherlands) for 30 min at RT. Double labeling of PLLP and BL-ICAM-1 was performed sequentially on thawed cryosections of PLLP-GFP HepG2 cells. In brief, sections were first labeled for ICAM-1 as above and fixed with 1% GLA for 5 min to stabilize the first immune reaction. After aldehyde quenching and protein blocking, sections were incubated for 30 min at RT with a rabbit anti-GFP antibody (A-6455, Invitrogen) followed by protein A conjugated to 10 nm gold particles (CMC) for 30 min. As negative controls, sections were incubated as indicated above but omitting either the bridge antibody or the anti-GFP antibody. After labeling, sections were stained with a mix of 1.8% methylcellulose and 0.4% uranyl acetate and visualized at 80 kV with a JEM 1010 (JEOL, Japan) electron microscope equipped with a F416 CMOS 4 K camera (TVIPS, Germany) or a JEOL JEM-1400 Flash electron microscope equipped with a One View CMOS 4 K camera (Gatan, United States).

### Protein extraction and western blot

Cell lysates were prepared using Laemmli buffer supplemented with a cocktail of protease inhibitors. The lysates were heated at 95 °C for 5 min and cleared by centrifugation at 14,000 rpm. for 5 min. The samples were loaded on acrylamide gels and transferred onto an Immobilon-PVDF membrane (Millipore), which was blocked in 5% milk and incubated overnight with the indicated primary antibody. Anti-rabbit or anti-mouse horseradish–peroxidase-(HRP)-conjugated secondary antibodies were then used and the antibody–protein complexes were visualized using ECL (GE-Healthcare). The bands intensities were quantified using Fiji software. For tissue extraction, mice were euthanized and 100 mg of liver from each animal were lysed in ice-cold 20 mM Tris–HCl pH 7.5, 5 mM EDTA, 0.2 mM EGTA using a polytron tissue homogenizer. Cell debris was removed by centrifugation for 5 min at 2000× *g* at 4 °C and supernatant protein concentration was measured with BCA protein assay (Thermo Fisher Scientific) and subjected to western blot analysis with the indicated antibodies.

### Biotinylation of ICAM-1-proximal proteins: BioID assay

To generate an expression plasmid containing the construct ICAM-1-BirA*, the sequence coding for GFP in the ICAM-1-GFP was substituted by the BirA* sequence [25] obtained from the Cav1-BirA* plasmid, kindly provided by Prof. I. Correas (Centro de Biología Molecular Severo Ochoa), with BsrGI and AgeI enzymes (New England Biolabs). The expression vector coding for ICAM-1-BirA* was transfected into HepG2 cells by electroporation and cell clones stably expressing ICAM-1-BirA* were selected with G-418 as previously described [5]. ICAM-1-BirA* HepG2 cells were cultured on 10 cm diameter plates and after 48 h, incubated with 50 μM biotin for 16 h, lysed and subjected to a pull–down assay of biotinylated proteins with neutravidin-agarose (Thermo Scientific) as previously described [49]. Lysates and pull–down pellets were analyzed by western blot.

### Immunoprecipitation assays

PLLP-GFP HepG2 cells were grown for 72 h and washed once in cold PBS and lysed in 400 μl of TNE buffer (50 mM Tris pH 7.4, 150 mM NaCl, 5 mM EDTA) containing 1% Triton-X100 and protease inhibitor cocktail. Lysates were incubated with 20 μl protein A-coated Sepharose (Sigma-Aldrich) previously conjugated with 5 μl of anti-GFP antibody by overnight incubation, for 3 h at 4 °C with agitation; rabbit IgG antibody was used as a control. Antibody-conjugated beads were rinsed in TNE + TX100 buffer five times and dried by aspiration. Immunoprecipitated proteins were eluted in 20 μl of Laemmli´s buffer and analyzed by western blot. For immunoprecipitation assays of the BL-ICAM-1 population, cells were basolaterally labeled with anti-ICAM-1 antibody as described, then incubated at 37 °C for the indicated times, and immediately lysed in TNE + TX100 buffer. The post-nuclear supernatant of the lysates was incubated with protein G-Sepharose for 3 h at 4 °C with agitation, rinsed and analyzed by western blot.

### Generation of HepG2 tumors

Athymic nude mice were purchased from Charles River and kept in standard conditions of 22 ± 2 °C temperature, 45–55% humidity, rate of 12/12 h light/dark cycle, and food and water ad libitum in the CBM Severo Ochoa animal facility. Before implantation, HepG2 cells were trypsinized, washed gently in PBS, and resuspended in Optimem. 1 × 10^6^/100 μl of HepG2 control or PLLP_KO cells were implanted subcutaneously on the left hind flank of each mouse. Tumor growth was monitored every 7 days by palpation until the tumor was measurable with calipers, whereupon the monitoring frequency increased to once every 2 days. Tumor size was calculated as its volume (mm^3^) using the formula [[length (mm) × width (mm)] × 0.5236] x width (mm). For ethical reasons, mice were sacrificed when tumor volume reached 1,500 mm^3^. Tumors were harvested, washed in PBS, fixed in 4% PFA for 24 h at 4 °C and processed for tissue immunofluorescence as described. All these animal experimentation procedures conformed to the European Guidelines for the Care and Use of Laboratory Animals (Directive 86/609) and were approved by the Ethical Committees for Animal Experimentation of Universidad Autónoma de Madrid and the Comunidad Autónoma de Madrid, Spain.

### Quantification and statistical analysis

Data are expressed as the mean plus standard deviation (SD) or mean plus the standard error of the mean (SEM). Student’s two-tailed *t* tests or two-way ANOVAs were used to establish the statistical significance (*p* < 0.05) of group differences, depending on the experiment. In all cases, data from at least three independent experiments were used. All calculations were performed using Prism 7 software.

## Supplementary Information

Below is the link to the electronic supplementary material.

Supplementary file1 (AVI 137 KB)

Supplementary file2 (AVI 135 KB)

Supplementary file3 (AVI 245 KB)

Supplementary file4 (AVI 330 KB)

Supplementary file5 (PDF 1376 KB)

## Data Availability

Data and material will be made available on reasonable request to the corresponding author.
