# Supplementary material for: Plasmolipin regulates basolateral-to-apical transcytosis of ICAM-1 and leukocyte adhesion in polarized hepatic epithelial cells
Source: Cell Mol Life Sci. 2022 Jan 9;79(1):61. doi: 10.1007/s00018-021-04095-z (PMC8743267; doi:10.1007/s00018-021-04095-z)
Supplement: Supplementary file 5 — Supplementary file5 (PDF 1376 KB) [file 18_2021_4095_MOESM5_ESM.pdf]

Supplemental Information

Plasmolipin regulates basolateral-to-apical transcytosis of ICAM-1 and leukocyte adhesion in polarized hepatic epithelial cells

Cacho-Navas et al.

Number of supplemental figures and figure legends: 7

Number of supplemental video legends: 4

SUPPLEMENTAL FIGURES AND FIGURE LEGENDS

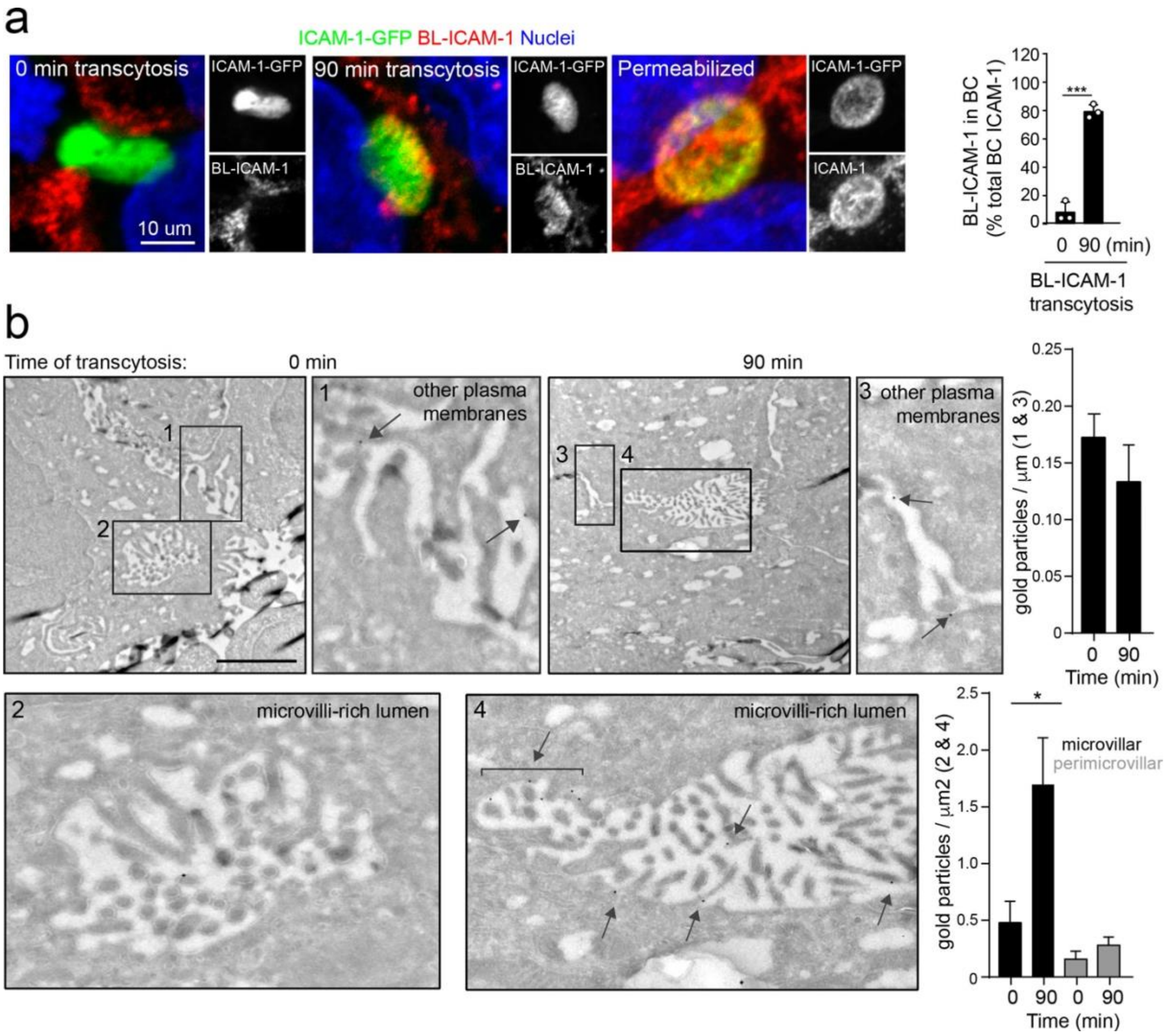

Figure S1. Related to Figure 1. **(a)** BL-ICAM-1 assays were performed as in Figure 1d in HepG2 stably expressing ICAM-1-GFP. Cells were fixed and stained for BL-ICAM-1 and nuclei. In parallel, ICAM-1-GFP cells were directly fixed and stained for ICAM-1. BL-ICAM-1 staining intensity in the transcytosis assay was expressed as percentage of the average staining intensity detected in canalicular ICAM-1 in permeabilized cells in each assay. ICAM-1-GFP was used to determine the BC area. 20 to 50 BCs were measured per experiment. Bars represent the mean ± SD. n=3. ***, $p<0.001$. Scale bar, 10 µm. Z-projections of at least 7 confocal planes of 0.6 µm thickness are shown **(b)** BL-ICAM-1 assays were performed as in Figure 1d and processed for electron microscopy as in Figure 1e. Top graph. Quantification of gold particles per µm of plasma membrane in cellular perimeter that do not correspond to microvilli rich cavities. Microvilli-rich cavities resembling BCs contained few gold particles at 0 min of transcytosis, which increased by more than three-fold upon 90 min of transcytosis. Bars represent the mean ± SD. 20 images were analyzed per condition. *, $p<0.05$. Scale bar, 2 µm

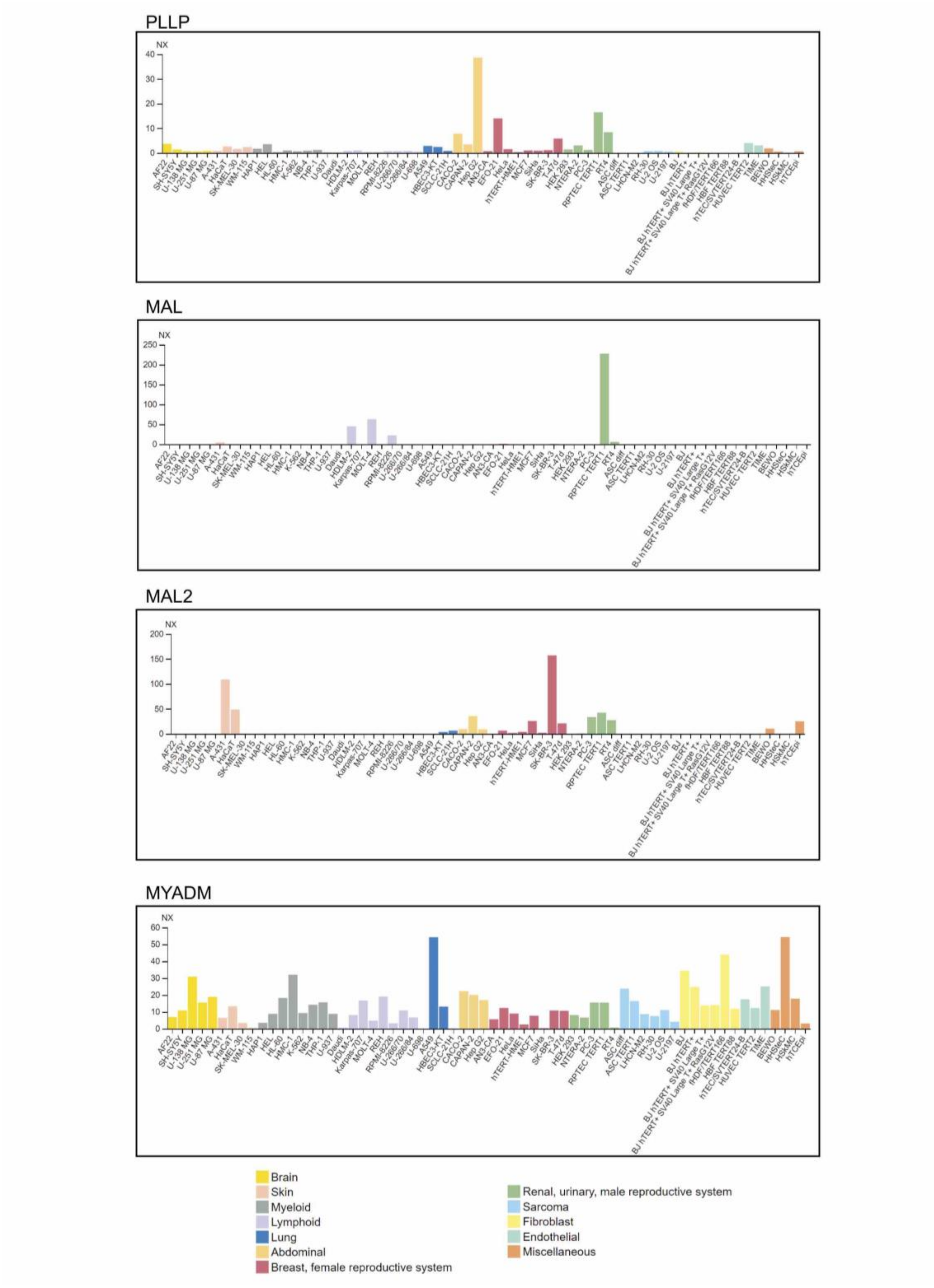

Figure S2. Related to Figure 2. mRNA expression levels of four MAL protein family members in the cell line panel from The *Human Protein Atlas* database (Uhlen et al., 2016).

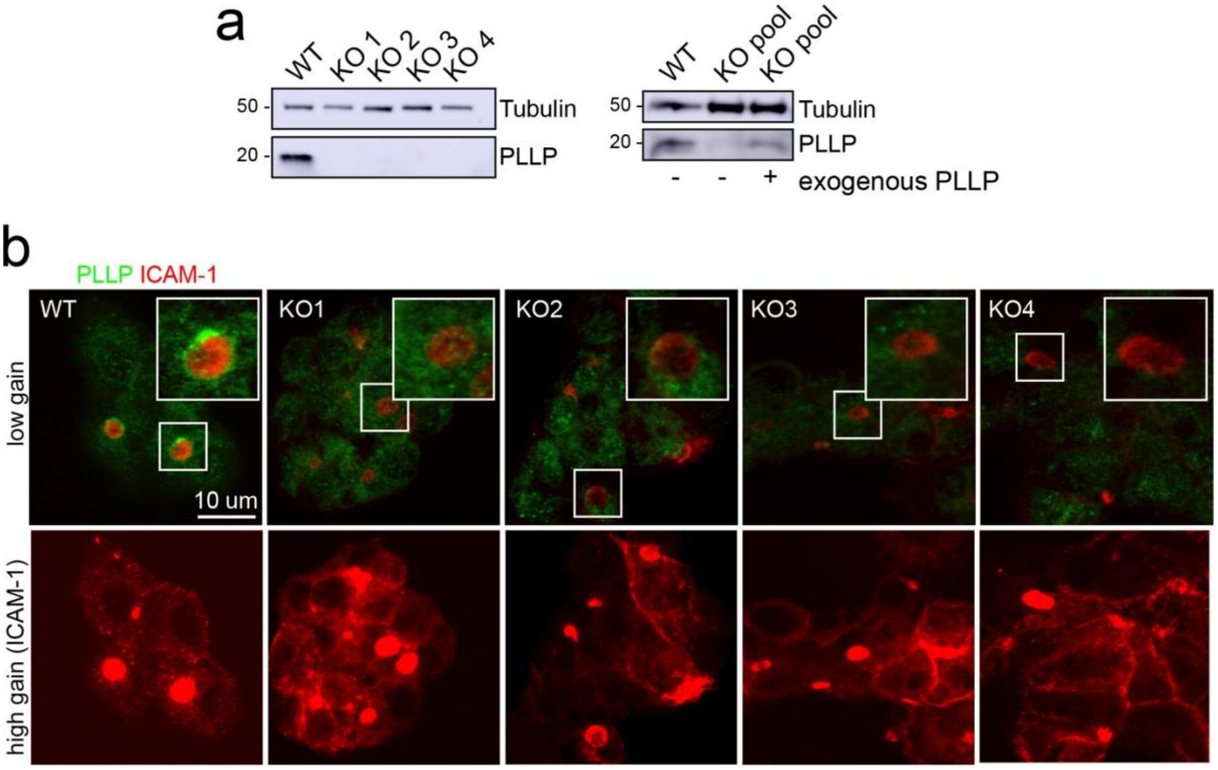

Figure S3. Related to Figure 2 and 4 . **(a,b)** The *PLLP* gene was edited using CRISPR/CAS9, and four PLLP_KO clones were selected and compared to parental HepG2 cells (WT). Clones were analyzed by western blot (a) and confocal immunofluorescence analysis (b) with a specific polyclonal antibody generated to the last 17 residues of human PLLP. (a) Left panels. Western blot of WT cells and PLLP_KO cell clones. Right panels. The clones were pooled and transiently transfected with an expression vector coding for human PLLP. The antibody specifically recognized a 20 KD band in transfected KO cells corresponding to exogenous PLLP expression. (b) Note that the pericanalicular staining with the anti-PLLP antibody disappears in PLLP_KO cells, although some background signal remains. ICAM-1 staining decreased in BC domains (top images) and increased its non-canalicular, basolateral distribution in the KO clones (bottom images) with respect to WT cells. Images were acquired in the confocal microscope with two levels of gain to show changes of ICAM-1 staining in canalicular (low gain) and non-canalicular (high gain) membrane domains (quantifications shown in Figure 4c-d) Scale bars, 10 μm. Z-projections of at least 10 confocal planes of 0.6 μm thickness are shown.

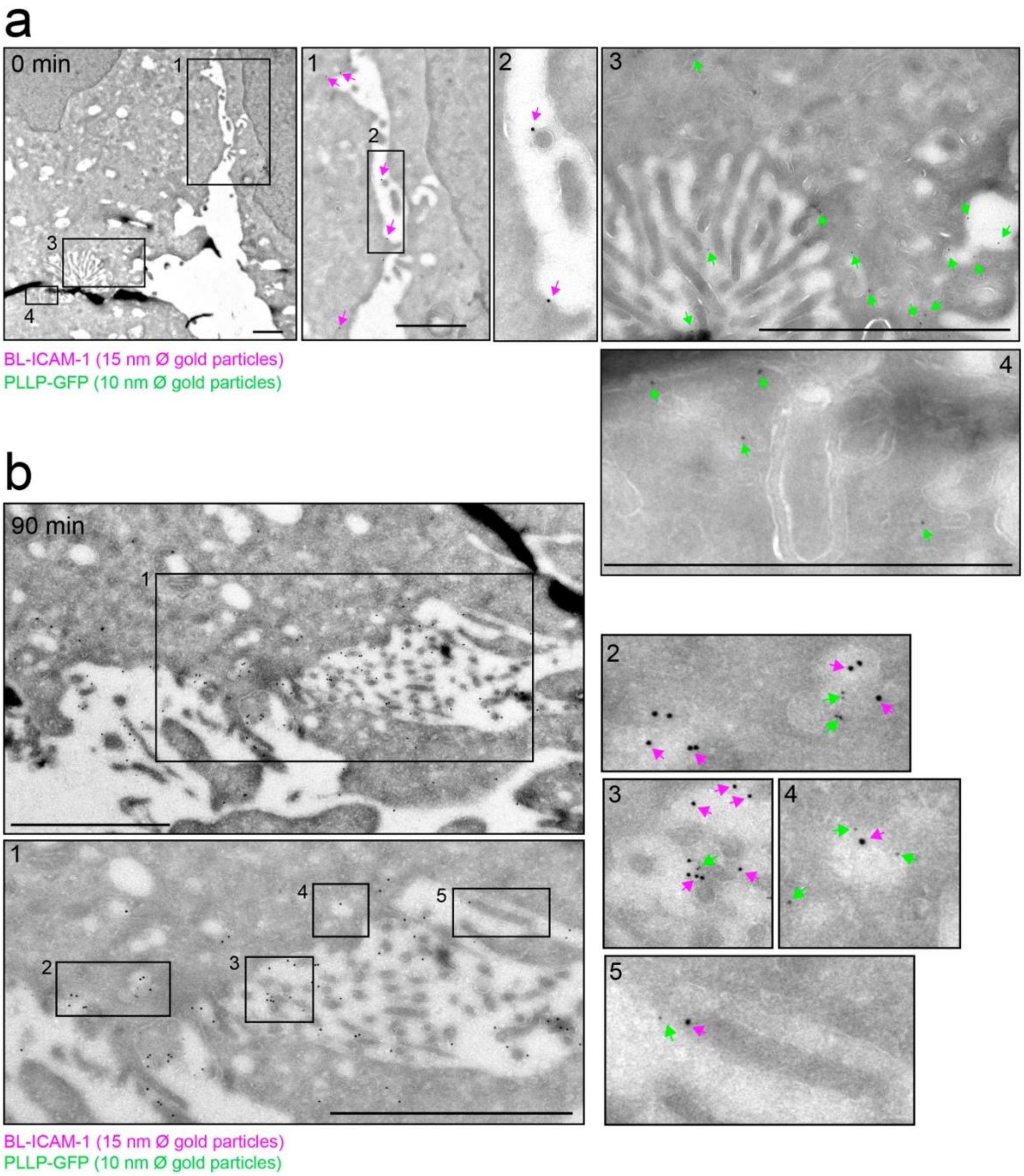

Figure S4. Double immunolocalization of BL-ICAM-1 and PLLP-GFP by transmission electron microscopy. ICAM-1 was basolaterally-labeled as in Figure 1d. Cells were then incubated at 37°C for 0 min **(a)** or 90 min **(b)**, and fixed following procedures compatible with immunolocalization by transmission electron microscopy. BL-ICAM-1 was detected with a rabbit anti-mouse antibody followed by protein A conjugated to 15-nm gold particle (purple arrows). Rabbit anti-GFP antibody was followed by incubation with a protein A conjugated to 10-nm gold particles (green arrows). Scale bars 2 μm.

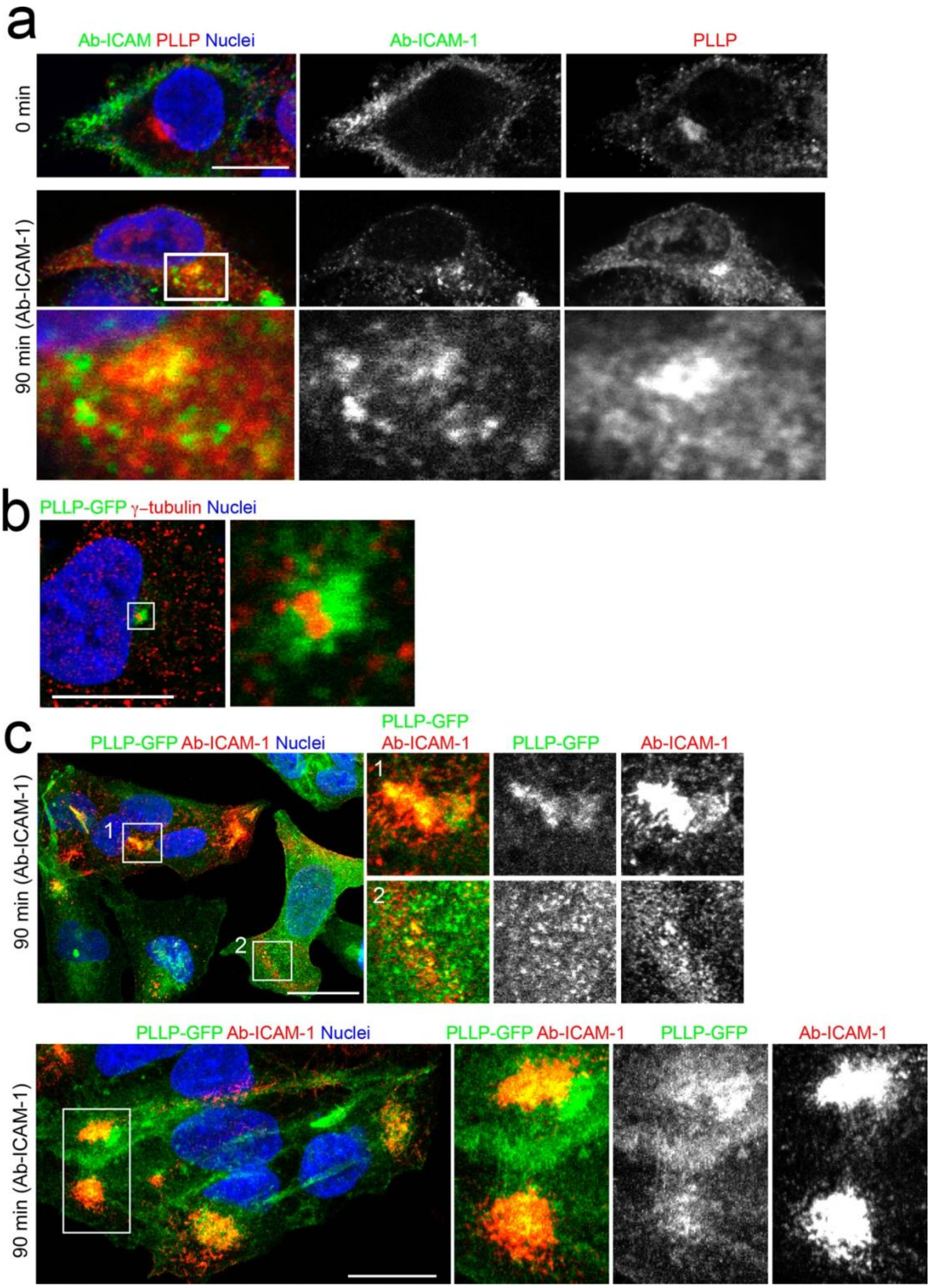
a
Ab-ICAM PLLP Nuclei
Ab-ICAM-1
PLLP
0 min
90 min (Ab-ICAM-1)
b
PLLP-GFP γ–tubulin Nuclei
c
PLLP-GFP Ab-ICAM-1 Nuclei
PLLP-GFP Ab-ICAM-1
PLLP-GFP
Ab-ICAM-1
1
2
90 min (Ab-ICAM-1)
PLLP-GFP Ab-ICAM-1 Nuclei
PLLP-GFP Ab-ICAM-1
PLLP-GFP
Ab-ICAM-1
90 min (Ab-ICAM-1)

Figure S5. Related to Figure 3. HepG2 cells were seeded at subconfluence for 24 h. ICAM-1 was labelled with a specific antibody in a manner similar to the basolateral labeling performed in polarized HepG2 cells in Figure 3c and e. The distribution of antibody labeled (ab)-ICAM-1 at the indicated times of trafficking was analyzed by confocal microscopy. **(a)** Intracellular aggregates with a vesicular pattern were observed at 90 min (bottom) but not at 0 min (top) of trafficking. These aggregates partially overlapped with endogenous PLLP in a compartment close to the nucleus (boxed area). Z-projections of at 6 confocal planes of 0.7 μm thickness are shown **(b)** Double staining between PLLP-GFP and the centrosomal marker γ-tubulin showing that a proportion of PLLP concentrates around centrosomes like some other proteins of the recycling compartment. A single confocal plane is shown. **(c)** Ab-ICAM-1 also accumulates in big clusters (top image, enlargement of boxed area 1, bottom image) and in small vesicular-like pattern (top image, enlargement of boxed area 2) after 90 min of trafficking. These two types of distribution partially overlapped with PLLP-GFP. Scale bars, 10 μm. Z-projections of at least 6 confocal planes of 0.7 μm thickness are shown.

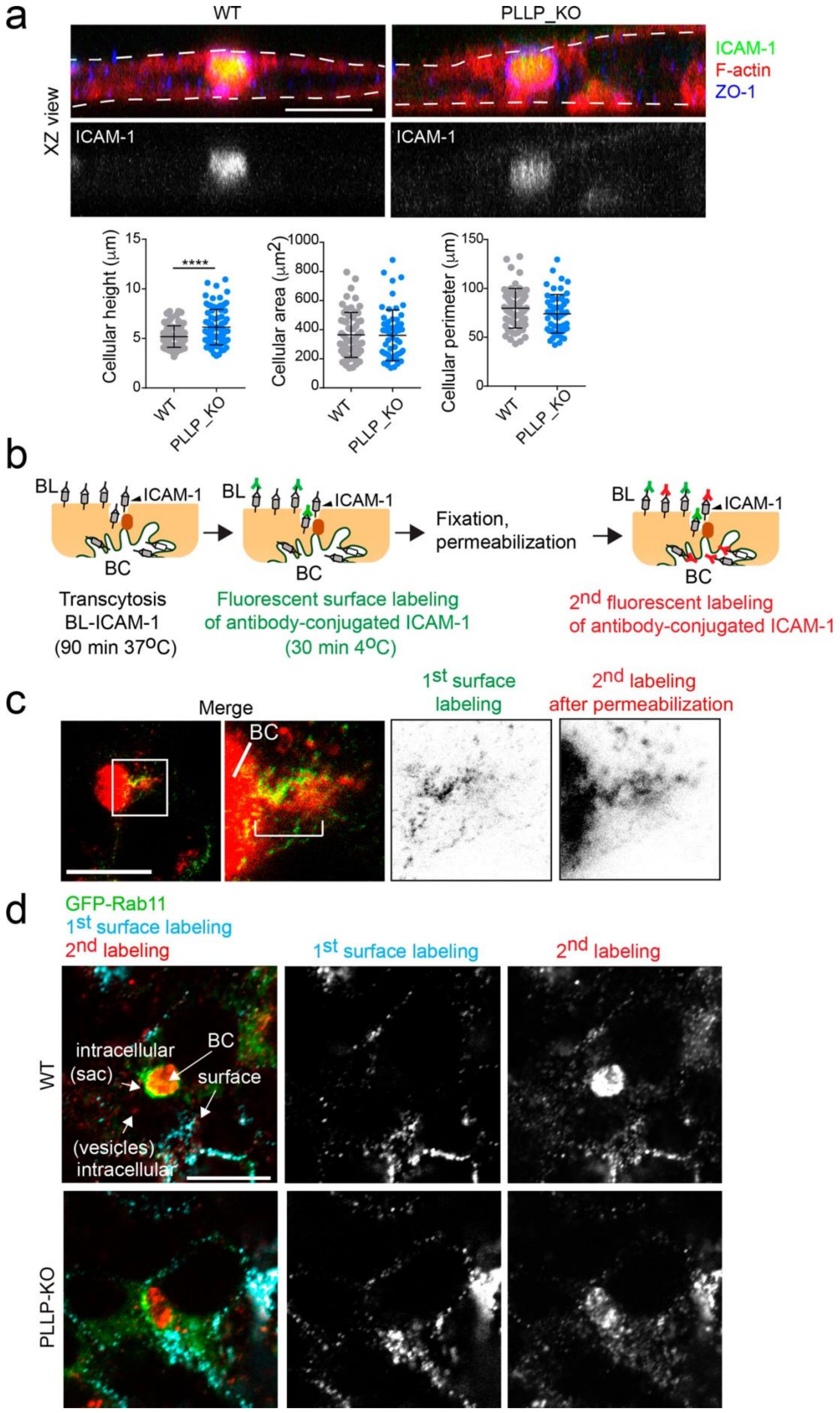
a
WT
PLLP_KO
XZ view
ICAM-1
F-actin
ZO-1
ICAM-1
ICAM-1
Cellular height (μm)
****
Cellular area (μm2)
Cellular perimeter (μm)
WT
PLLP_KO
b
BL
ICAM-1
BC
Transcytosis
BL-ICAM-1
(90 min 37oC)
Fluorescent surface labeling
of antibody-conjugated ICAM-1
(30 min 4oC)
Fixation,
permeabilization
2nd fluorescent labeling
of antibody-conjugated ICAM-1
c
Merge
1st surface labeling
2nd labeling after permeabilization
BC
d
GFP-Rab11
1st surface labeling
2nd labeling
WT
intracellular (sac)
BC
surface
(vesicles) intracellular
PLLP-KO

Figure S6. Related to Figure 4. **(a)** Confocal analysis of cellular height, spreading area and perimeter in WT and PLLP_KO HepG2 cells. 75 and 88 Z-stacks, respectively, composed by at least 16 confocal planes, were quantified for each cell type. The triple staining of F-actin, ICAM-1, and ZO-1 was used for quantification. **(b)** Schematic representation of the sequential incubation with secondary antibodies that bind BL ICAM-1 performed to discriminate between surface and internalized BL-ICAM-1 at 90 min of transcitosis. The incubation with a first secondary antibody at 4°C before fixation specifically labels surface BL-ICAM-1 that has not been internalized. After fixation and permeabilization, incubation with a second secondary antibody conjugated with a different fluorophore labels the internalized BL-ICAM-1 and part of surface BL-ICAM-1. **(c)** Comparison between the two secondary-antibody distributions enables discrimination between internalized and surface ICAM-1 populations. Super-resolution confocal microscopy (STED) of BL-ICAM-1 after 90 min of transcytosis. The bracket points at regions of transition between surface and internalized ICAM-1. Note that the BC is not labeled by the first secondary antibody added prior fixation and permeabilization. **(d)** Transcytosis and sequential incubation of secondary antibodies to discriminate surface from internalized BL-ICAM-1 in WT and PLLP_KO cells expressing GFP-Rab11. After image acquisition, surface fluorescence intensity was subtracted from the channel corresponding to the staining performed after permeabilization, so that population of internalized BL-ICAM-1 in the SAC (identified by colocalization with GFP-Rab11) and in other intracellular vesicular domains could be quantified. Note that the representative image shows cells with a slightly greater than average surface distribution of BL-ICAM-1, in order to better illustrate the differences between surface and total labelling of BL-ICAM-1. Scale bars, 10 μm.

a

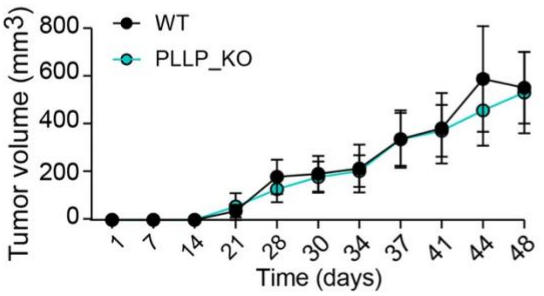

b

Injected Cells	Mouse	Harvesting-Appearance (days)	Infiltration in HepG2 tumor	Macrophage /μm2
WT cells	1	25	-	
	2	28	-	
	3	21	-	
	4	25	-	
	5	25	+	1.107e-04
	6	-	-	
	7	-	-	
	8	28	N/A	
	9	-	-	
	10	25	N/A	
	11	25	-	
	12	25	N/A	
PLLP_KO cells	1	28	N/A	
	2	25	+++	8.154e-04
	3	25	N/A	
	4	-	-	
	5	28	N/A	
	6	28	N/A	
	7	25	N/A	
	8	-	-	
	9	20	+	3.459e-05
	10	28	+++	7.609e-04
	11	25	++	1.297e-04
	12	-	-	

N/A = not available

Figure S7. Related to Figure 6e. **(a)** Average tumor volume was calculated from the average tumor volume in each animal at the indicated times. Graphs show the mean ± SD. n= 12 mice per tumor cell type. **(b)** Analysis of macrophage infiltration. Note that infiltrated macrophages were detected in only one tumor generated by WT cells, so statistical analysis could not be performed. Some tumors rapidly collapsed and disaggregated after isolation and could not be processed for IHQ and confocal analysis (N/A).

SUPPLEMENTAL VIDEO LEGENDS

Video S1. Time-lapse confocal microscopy of fluorescent BL-ICAM-1 translocation to the BC in polarized cells expressing PLLP-GFP. Images were acquired at 10 min intervals for 100 min and displayed at 2 frames per second.

Video S2. Time-lapse confocal microscopy of fluorescent BL-ICAM-1 translocation to the BC in which emissions of PLLP-positive tubular structures are detected. Images were acquired at 10 min intervals for 100 min and displayed at 2 frames per second.

Video S3. Time-lapse confocal microscopy of fluorescent BL-ICAM-1 translocation to the BC in polarized WT HepG2 cells. Images were acquired at 10 min intervals for 90 min and displayed at 2 frames per second.

Video S4. Time-lapse confocal microscopy of fluorescent BL-ICAM-1 translocation to the BC in polarized PLLP_KO HepG2 cells. Images were acquired at 10 min intervals for 90 min and displayed at 2 frames per second.
